# The Community Structures of Prokaryotes and Fungi in Mountain Pasture Soils are Highly Correlated and Primarily Influenced by pH

**DOI:** 10.3389/fmicb.2015.01321

**Published:** 2015-11-27

**Authors:** Anders Lanzén, Lur Epelde, Carlos Garbisu, Mikel Anza, Iker Martín-Sánchez, Fernando Blanco, Iker Mijangos

**Affiliations:** Soil Microbial Ecology Group, Department of Conservation of Natural Resources, NEIKER-TecnaliaDerio, Spain

**Keywords:** soil microbial communities, soil properties, pasture management, grassland soil, biodiversity, microbial diversity, belowground interactions

## Abstract

Traditionally, conservation and management of mountain pastures has been managed solely on the basis of visible biota. However, microorganisms play a vital role for the functioning of the soil ecosystem and, hence, pasture sustainability. Here, we studied the links between soil microbial (belowground) community structure (using amplicon sequencing of prokaryotes and fungi), other soil physicochemical and biological properties and, finally, a variety of pasture management practices. To this aim, during two consecutive years, we studied 104 environmental sites characterized by contrasting elevation, habitats, bedrock, and pasture management; located in or near Gorbeia Natural Park (Basque Country/Spain). Soil pH was found to be one of the most important factors in structuring soil microbial diversity. Interestingly, we observed a striking correlation between prokaryotic, fungal and macrofauna diversity, likely caused by interactions between these life forms. Further studies are needed to better understand such interactions and target the influence of different management practices on the soil microbial community, in face of the significant heterogeneity present. However, clearing of bushes altered microbial community structure, and in sites with calcareous bedrock also the use of herbicide vs. mechanical clearing of ferns.

## Introduction

Mountain areas cover approximately 27% of the world's terrestrial area and play important ecological roles globally and locally, including carbon sequestration and retention of biodiversity (UNEP World Conservation Monitoring Centre, 2002; IPCC, 2007). The maintenance of these ecosystems is also vital for mountain ranching, an activity of significant socio-economic and cultural importance to mountainous and semi-mountainous regions in Europe and elsewhere. In addition to contemporary climate change, with its disproportionate effect on mountain areas (IPCC, 2007), poor management such as over-grazing, fertilization or aggressive use of herbicides can adversely impact the functioning of these agro-ecosystems (Tilman et al., 2001; Foley et al., 2005; Wakelin et al., 2009). Similarly, a cessation of activity in areas with a long pastoral tradition is causing a loss in biodiversity as shrubs spread (Montalvo et al., 1993; Watkinson and Ormerod, 2001). Therefore, it is essential to promote agricultural practices that increase forage yield and nutritive value while preserving biodiversity and pastoral ecosystem functioning and sustainability (Mijangos et al., 2010). A simple example is clearing of shrubs, frequently carried out to improve pasture quality.

Traditionally, conservation and management of mountain pastures has been managed solely on the basis of visible biota. However, microorganisms (including prokaryotes and eukaryotes) play vital roles in the soil ecosystem, and influence plant community composition and productivity, together with other factors relevant to pastoral production systems (Barrios, 2007). Thus, in terrestrial ecosystems, it is necessary to also take the soil microbiome into account, to better understand essential processes such as the dynamics of plant production, the conservation of biodiversity, the maintenance of soil health, the capacity of the soil to sequester carbon, etc. (Maron et al., 2011). Recently, technological advancements (mainly DNA sequencing) have opened up for large-scale studies of microbial community structure. However, relatively little is yet known about how microbial communities are influenced by pastoral management practices or a cessation of such activity.

The most significant challenge faced by microbial ecology, in spite of recent methodological advancements, is probably the enormous complexity and heterogeneity of soil and its micro-habitats (Vos et al., 2013). The soil microbial foodweb is complex and largely uncharacterized with estimates of total number of microbial “species” present in 1 g of soil ranging from thousands to 10^7^ (as reviewed in Delmont et al., 2011). Different studies have indicated pH as one of the most important determinants of microbial alpha diversity and composition (Lauber et al., 2008; Rousk et al., 2010; Kuramae et al., 2012), together with other factors including carbon and nutrient availability (Fierer et al., 2007; Shange et al., 2012; Tripathi et al., 2012; Zhalnina et al., 2014). Depending on study design and the heterogeneity among sites studied, such soil properties may have a stronger influence on microbial communities compared to land use and agricultural practices as such (Kuramae et al., 2012). A diversity of land-use patterns, fertilization and other agricultural practices exist, often varying across conventional farming systems, adding further to the complexity of assessing their influence on soil microbial communities (Hartmann et al., 2015). In this respect, it is important to also consider the indirect effects of agricultural practices via soil properties (Lauber et al., 2008).

The project SOIL-MONTANA (http://www.soilmontana.com) was devised to standardize and integrate various physicochemical and biological soil parameters as well as biodiversity of macro- and micro-biota into management of mountain and valley pastures. For this purpose, Agroecosystem Health Cards (AHCs) were designed (Mijangos et al., 2013; available as Supplementary Presentation 1) and utilized to characterize 104 environmental sites (including biological replicates) during two consecutive years, in collaboration with local farmers. All sites were located in Gorbeia Natural Park and its immediate surroundings, characterized by contrasting elevation, habitats, bedrock (parent material), and pastoral practices.

Gorbeia is located in the mostly mountainous Basque Autonomous Community (Northern Spain). It is a protected area that has long-standing pastoral tradition with evidence of activity since the neolithic (Zapata et al., 2004) and has been designated as a Site of Community Importance by the European Commission. As such, it is a good example of an area under threat from progressive cessation of grazing activities due to economic factors and a lack of generational replacement (Lasanta-Martínez et al., 2005).

The comprehensive data collection carried out in SOIL-MONTANA served to evaluate AHCs as a tool for assessment of agroecosystem sustainability and influence of pastoral management practices, while also increasing ecological and scientific awareness of local farmers and stakeholders (Mijangos et al., 2013). Specifically, the impacts of fertilization, liming of relatively acid mountain soils, clearing of bushes in semi-abandoned pastures, and chemical vs. mechanical clearing of ferns were evaluated, as well as differences between land use (harvested vs. pastured valley grassland; see Table 1). Here, we expand this assessment by studying belowground community structure in-depth. For this purpose, the small-subunit ribosomal RNA gene (16S rRNA) and the internal transcribed spacer (ITS) were amplified from community DNA as taxonomic markers for prokaryotes and fungi, respectively, and sequenced using Illumina MiSeq. We note that our approach may target large as well as microbial fungi, possibly even from different life stages of the same species, but here we refer to the resulting belowground community structure as “microbial” for simplicity.

**Table 1:** **Overview of sample site characteristics**.

**Group**	Elevation zone^*^	**Treatment**	Land-use^**^	**Bedrock**	**Sites 2013**	**Sites 2014**
VI1	V	Inorganic fertilizer	P	Calcareous	4	4
VI2	V	“	H	Mixed	4	4
VI3	V	“	X	Mixed	4	4
VO1	V	Organic fertilizer	P	Calcareous	4	4
VO2	V	“	H	Mixed	4	4
VO3	V	“	X	Mixed	4	4
VE1	V	Liming	P	Siliceous	4	4
VE2	V	“	H	Siliceous	4	4
VE3	V	“	X	Siliceous	4	4
VNE1	V	No liming	P	Siliceous	4	6
VNE2	V	“	H	Siliceous	4	4
VNE3	V	“	X	Siliceous	4	4
MP1	HM	Addition of phosphorus	P	Siliceous	4	4
MP2	HM	“	P	Calcareous	4	4
MP3	HM	“	P	Siliceous	4	4
MNP1	HM	No addition of P (negative control)	P	Siliceous	8	4
MNP2	HM	“	P	Calcareous	8	4
MNP3	HM	“	P	Siliceous	3	4
MDC	HM	Clearance (year 1)	P	Siliceous	4	4
MDL	HM	Clearance (year 3)	P	Siliceous	4	4
MDM	HM	Clearance (year 5)	P	Siliceous	4	4
HH1	LM	Chemical fern control	P	Siliceous	4	4
HH2	LM	“	P	Calcareous	4	4
HNH1	LM	Mechanical fern control	P	Siliceous	4	4
HNH2	LM	“	P	Calcareous	4	4

* *V, Valley (240–410 m elevation); LM, Low Mountain (630–720 m); HM, High Mountain (890–100 m)*.

** *P, Grazed pasture; H, Harvested; X, Mixed (grazed during winter only)*.

**Figure 1:**
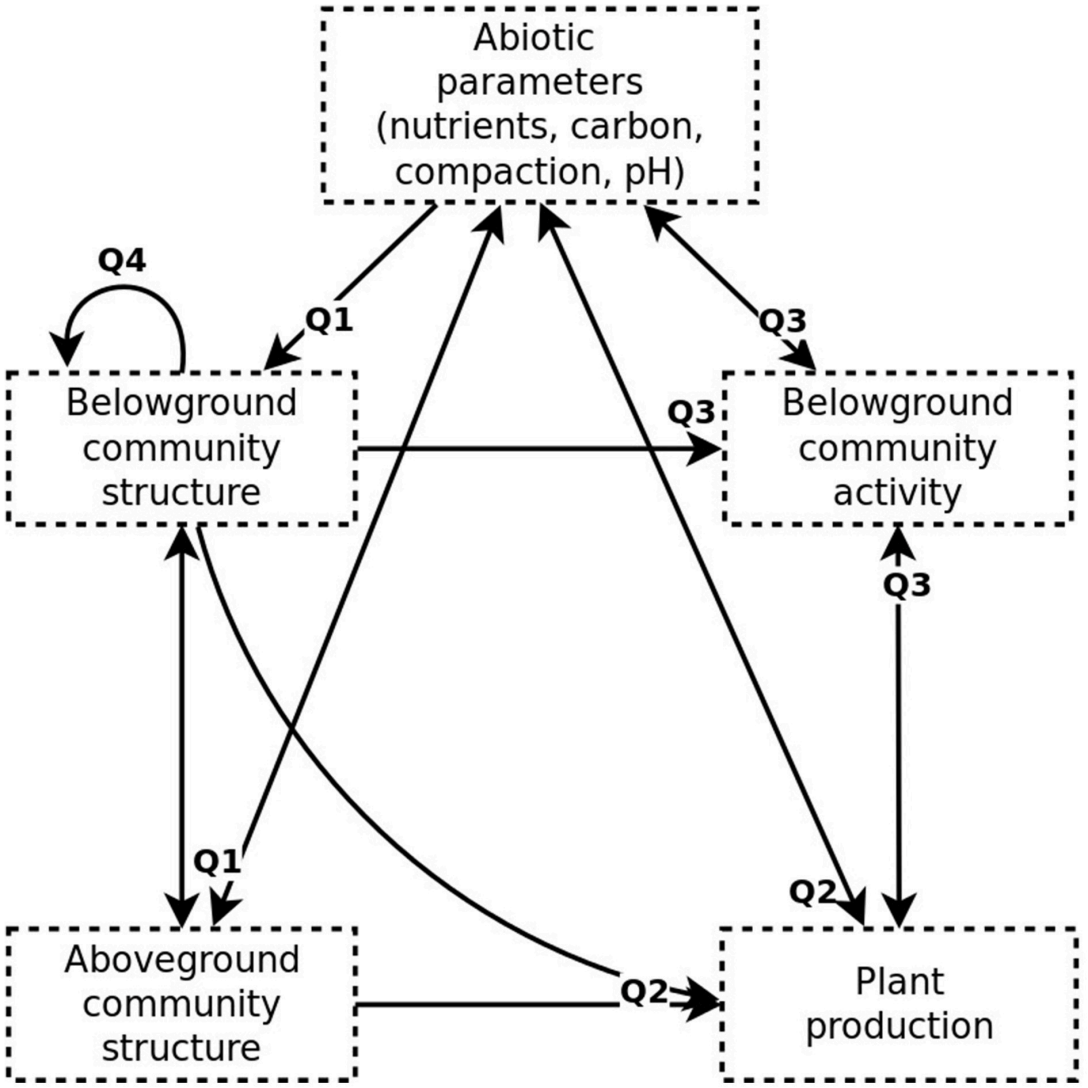
**Simplified theoretical meta-model of the grassland ecosystem studied**. Causal links are illustrated by arrows and questions associated to particular links indicated.

We propose a theoretical meta-model illustrating how the dynamics of the studied ecosystem is determined by an interplay of soil parameters, above- and belowground community structure and activity (see Figure 1). Further, we expect a high degree of interactions between taxa, and abundances of such interacting to be correlated across time or space. This may also be reflected in correlation of diversity indices of different groups of organisms that show a high degree of interactions and thus depend on each other.

We focus on five general questions regarding biodiversity, ecosystem function and the impact of management practices in the studied area (also illustrated in Figure 1):

(Q1) How do abiotic soil parameters influence below- and aboveground community structure? Here we consider nutrients, carbon, soil compaction and pH.
(Q2) How do abiotic parameters, together with below- and aboveground community structure, influence plant production?
(Q3) How do plant production, abiotic parameters and belowground community structure influence belowground community activity?
(Q4) Can we identify interactions between belowground taxa that contribute to structuring the belowground community?
(Q5) How may different pastoral and agricultural management practices influence ecosystem function?

Out of the above, Q5 is perhaps the most relevant for improving conservation and agricultural guidelines. However, a basic understanding of the dynamics of the ecosystem studied (Q1–4) is necessary to answer it, since changes in land use and management practices may influence all of the ecosystem components, which depend on each other through causal links including feedbacks (Figure 1). These feedbacks, in addition to its biodiversity and heterogeneity, illustrates the notorious complexity of the soil ecosystem (Barrios, 2007), and exposes our questions as somewhat naïve. Nonetheless, we show that they can be partially answered, by using structural equation modeling (SEM), multivariate statistics, ANOVA and rank-correlation analysis. Thus, we suggest trophic or mutualistic interactions, as well as factors that appeared to influence biodiversity, belowground activity and plant productivity.

## Materials and methods

### Study sites, treatments, and sample collection

The study included soil samples from 104 sites from Gorbeia Natural Park and its proximity, organized into 25 groups representing 3–8 sample replicates each from different combinations of land-use, agricultural practices and bedrock (calcareous vs. siliceous; see Table 1). Groups representing different treatments were located at three different elevation ranges: valley sites (*V*) at 240–410 m, low mountain sites (*LM*) at 630–720 m and high mountain sites (*HM*) at 890–1100 m. Valley sites were also characterized by three different land uses: year-round grazed pastures (*P*), mixed (*X*: grazed during winter and early spring and harvested for forage during the most productive season), and harvested non-grazed sites (*H*). Mountain sites were grazed except during winter.

Experimental agricultural treatments are listed in Table 1 and included fertilization (*V* and *HM* sites), liming (*V*) and chemical vs. mechanical removal of ferns (*LM*). In addition, bushes were cleared from a 40 ha area during the end of 2012 from which one group of samples was collected, in order to investigate the effect of cessation of the practice of clearing mountain pasture land from bushes. For that reason, control samples were also collected from two areas cleared previously (3 and 5 years before the first sampling). For each type of land-use in *V*, one group of sites was treated with organic fertilizer (cattle or sheep manure, 29–35 tons ha^−1^ applied during autumn) one with mineral NPK-fertilizer (NH_4_NO_3_ + P_2_O_5_ + ClK, 200–250+50–60+275–350 units ha^−1^, applied during spring), one subjected to liming (1500 kg Ca(OH)_2_ ha^−1^; applied during March, 3 months prior to sampling), and a fourth used as negative control for liming. Three groups of *HM* sites were subjected to fertilization with organic rock phosphate (192 kg ha^−1^ of 26.5% P_2_O_5_ and 29% CaO, applied during spring) each representing different habitat types (see Table 1). *Nardus stricta* (matgrass) was present in two of these sites, representing habitats of conservation priority in the European Union. In the sites evaluating fern control, chemical treatment was carried out by applying 5 l ha^−1^ Asulox® herbicide (40% methyl sulfonyl carbamate), and mechanical control by chain brush cutter.

From each site, soil physicochemical parameters were determined and samples collected during 2013 as well as 2014 (15 April 15–2 October depending on treatment; see Table S1 for sampling dates and resulting metadata). Topsoil from 0–10 cm was collected using a core soil sampler (25 mm diameter) and sieved to <2 mm and before measurements of physicochemical parameters, air-dried at ambient temperature until constant weight. For biological parameters, soils were instead stored fresh at 4°C for a maximum of 2 months until analysis. Sub-samples for molecular analysis were stored at −20°C.

### Parameters measured using agroecosystem health cards

Physicochemical and biological parameters were measured as described in the AHCs (Table 2; Supplementary Presentation 1). Basal respiration (indicating total belowground activity) was measured according to ISO standard 16072:2002^1^ and substrate-induced respiration (indicating potential belowground activity) according to ISO 17155:2002^2^. Aluminum saturation, pH, total nitrogen (N), phosphorus content (Olsen P) and extractable potassium content (K) was measured according to MAPA (1994) as further described in Supplementary Presentation 1.

**Table 2:** **Overview of considered parameters, measured according to Agroecosystem Health Cards (AHCs; see Supplementary Presentation 1)**.

**Ecosystem service**	**Parameter**	**Unit**	**AHC parameter**	**Indicator of (Figure 1)**
Pasture production	Fresh weight	kg m^−2^ per year	1.1 basic	Aboveground activity
	Dry weight	t ha^−1^ per year	1.1 advanced	“
Biodiversity conservation	Plant richness	(Number)	2.1 basic	Aboveground community structure (c. s.)
	Plant shannon diversity (H')	(Number)	2.1 advanced	“
	Types of macrofauna	(Number)	2.3 basic	Belowground c. s.
	Fungal and prokaryotic genetic diversity		2.7 advanced	“
Soil conservation	Earthworm abundance	individuals m^−2^	3.1 basic	Belowground c. s. and activity
	Induced respiration	mg C-CO_2_ kg^−1^ h^−1^	3.2 advanced	“
	Basal respiration	mg C-CO_2_ kg^−1^ h^−1^	3.1 advanced	Belowground activity
	Penetrability	Cm	3.2 basic	Abiotic: compaction
	Infiltration time	Minutes	3.4 basic	“
	Compaction	MPa	3.4 advanced	“
	Aluminum saturation	%	3.5 advanced	Abiotic: acidity
	pH		3.5 advanced	“
	Total N	%	3.6 advanced	Abiotic: nutrients
	Olsen P	mg kg^−1^	3.7 advanced	“
	Extractable K	mg kg^−1^	3.8 advanced	“
Carbon sequestration	Root abundance	(index: 1–10)	4.1 basic	(not in model)
	CO2 soil emission	g CO_2_ m^−1^ h^−1^	4.1 advanced	Belowground activity
	Soil color	(index: 1–10)	4.2 basic	Abiotic: carbon
	Soil Organic matter (SOM)	%	4.2 advanced	Abiotic: carbon

### DNA extraction, amplification, and sequencing

DNA extraction was carried out from aliquots corresponding to 0.25 g of dry-weight soil from all samples using PowerSoil DNA Isolation kits (Mo-Bio Laboratories, Carlsbad CA), following the manufacturer's instructions. Prior to DNA extraction, samples were washed with 120 mM K_2_HPO_4_ to remove extracellular DNA (Kowalchuk et al., 1997).

For preparation of 16S rRNA amplicon libraries, we used 515F (CTGNCAGCMGCCGCGGTAA) and 806R (GGACTACHVGGGTWTCTAAT) modified from Caporaso et al. (2012), targeting the V4 hypervariable region. For fungal ITS, we used ITS1F (CTTGGTCATTTAGAGGAAGTAA) (Gardes and Bruns, 1993) and ITS2R (GCTGCGTTCTTCATCGATGC) targeting the ITS1 region (White et al., 1990). Dual-indexed adapter linked amplicons were prepared using a proprietary protocol based on Caporaso et al. (2012) at StarSEQ GmbH, Mainz, Germany. Sequencing was carried out using an Illumina MiSeq with the V2 kit and pair-ended 2 × 250 nt dual-index sequencing at StarSeq, Mainz, Germany. Sequence data has been deposited to the European Nucleotide Archive with study accession PRJEB9654.

### Sequence data processing and taxonomic classification

Read-pairs from 16S rRNA amplicons were quality-filtered and overlapped using *usearch* (options *fastq_truncqual* = *10, fastq_maxdiff* = *5, fastq_maxee* = *0.5*) and truncated to a length of 253 nt representing the expected amplicon length between primers (Edgar, 2013). ITS read-pairs were trimmed to remove the reverse primer using *cutadapt* (Martin, 2011) before being overlapped using the same parameters, without truncation, since large numbers of “staggered” pairs were encountered and amplicon lengths found to vary considerably. All quality-filtered overlapped sequences from 16S rRNA and ITS amplicons were merged across datasets and clustered into OTUs at 97% sequence similarity using *vsearch* (Rognes et al., 2015). Clustering included de-replication, sorting by abundance (descending and not retaining singletons), then clustering into OTUs at 97% sequence similarity and finally chimera filtered using the *uchime* reference-based method using the ChimeraSlayer reference database, followed by *de novo* filtering.

Representative prokaryotic and fungal OTU sequences were aligned to the SilvaMod v106 and UNITE reference databases, respectively, using *blastn* (v.2.2.25+ task megablast) and taxonomically classified using CREST with default parameters (Lanzén et al., 2012). The resulting taxon distribution was studied at order rank as determined by CREST for the fungal ITS datasets, and at family rank for prokaryotic taxa of the 16S rRNA datasets. Taxa of lower ranks detected that lacked child nodes at order or family level were manually included into the respective dataset. Relative taxon abundances derived by CREST were used in further analysis (the sum of read abundances mapped to OTUs classified to a particular taxon or its child nodes in a particular sample classified, divided by total sample reads) and plotted using the R package *ggplot2* (Wickham, 2009). This approach was motivated by the fact that sequence-based relative abundance has been demonstrated to provide meaningful semi-quantitative information when comparing community structure between samples (Pilloni et al., 2012), in spite of being affected by issues such as ribosomal copy number variability and preferential amplification.

### Structural equation modeling (SEM) and statistical analyses

Multivariate statistics, calculation of diversity indices and visualization was performed using the R *vegan* package (Oksanen et al., 2015). Rarefied richness values estimating the expected richness at the lowest sample-specific sequencing depth were used to compensate for the considerable variation in read numbers. OTU distribution across samples was transformed into relative abundances using the function *decostand*. Community composition between samples was thereafter compared using Bray-Curtis dissimilarities. Non-metric multidimensional scaling (NMDS) was carried out using *metaMDS*. Correlation analyses between continuous parameters or taxon abundances; with factors such as treatment (Q5), land-use (Q5), bedrock and elevation zone; were determined using group-wise ANOVA and Tukey's Range Test (Honest Significant Difference). All significance estimates calculated using *envfit* or ANOVA were subjected to Bonferroni correction and not reported unless *p* < 0.05 after correction.

ANOSIM (analysis of similarity) was also carried out based on Bray-Curtis dissimilarities in order to evaluate the effect of overall community structure of different site types (including land-use) or agricultural practices / treatments (Q5). Due to the strongly different characteristics of mountain (*M*) and valley (*V*) sites, all ANOVA and ANOSIM analyses were also carried out group-wise for the two corresponding subsets where appropriate, and are not reported unless verified in either *M* or *V*, in order to avoid false conclusions based on sample selection.

A SEM was developed based on the theoretical meta-model constructed (Figure 1) and questions Q1–Q3 listed in the Introduction. In order to form a directed acyclic graph (DAG), solvable using a maximum likelihood approach, this model ignores several fundamentally bidirectional causal links, such as those between biological activity and abiotic parameters. Further, the SEM does not incorporate the highly multidimensional data available, such as belowground community OTU or taxon composition. Instead, it uses a latent variable combining Shannon estimates (H') of prokaryotic and fungal diversity, thus taking into account both species richness and evenness; as well as macrofauna types. The SEM was solved for all complete data available, as well as the *M* subset, using function *sem* of the R package lavaan (Rosseel, 2012). Due to insufficient data size, it could not be solved for *V*. Variances for latent variables were fixed to 1 and exogenous variables were assumed to be independent. Nutrient concentrations were log-transformed and compaction measured standardized prior to model fitting.

To verify and extend the results from the SEM analysis, targeting effects of abiotic parameters on taxon composition rather than diversity (Q1), correlations between explanatory continuous variables and taxon abundances were determined using Kendall's rank correlation. Bonferroni correction and group-wise confirmation in the *M* and *V* subsets was also carried out for these analyses as described above. Further, Pearson correlations were determined between diversity estimates of different groups (prokaryotes, fungi and macrofauna; Q4).

Co-occurrence network analysis (Q4) based on all taxa (merging ITS and 16S rRNA datasets) with average relative abundance above 0.01% was performed with the R package *igraph* (Csardi and Nepusz, 2006) using Kendall rank correlation. All correlations resulting in tau coefficients above 0.7 or below -0.7 were visualized as edges and taxa with at least one edge included in further analysis.

## Results

### Community diversity and taxonomic structure

Sequencing resulted in over 26 million quality-filtered small-subunit ribosomal RNA gene (16S) amplicon sequences and approximately the same number for ITS. These were distributed over 31,470 and 17,639 16S and ITS OTUs, respectively. Table S2 provides an overview of read counts and diversity estimates for all samples. The number of reads correlated significantly with total OTU richness (before rarefaction; *p* = 3E-7, ρ = 0.3 for 16S; *p* < 2E-16, ρ = 0.9 for ITS). This indicated that our sequencing effort was insufficient to obtain full coverage of the diversity and rarefied richness estimetes were instead used to compare alpha-diversity between samples. Shannon diversity estimates, known to be more robust to sequencing effort did not show corresponding correlations.

**Figure 2:**
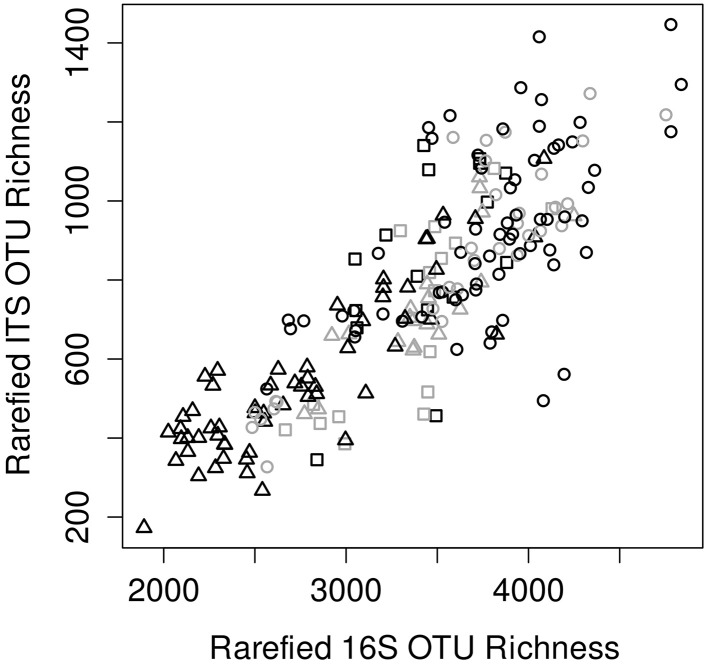
**Comparison of prokaryotic (16S) vs. fungal (ITS) rarefied richness estimates between samples**. Valley samples are symbolized by circles, low mountain by squares and high mountain by triangles. Samples with siliceous bedrock are colored black while those from calcareous are colored gray.

Prokaryotic diversity showed strong linear correlation with fungal diversity in terms of rarefied OTU richness (*p* < 2E-16, ρ = 0.84; see Figure 2), Shannon entropy (H'; *p* < 2E-16, ρ = 0.61), and Pielou's evenness (*p* = 3E-5, ρ = 0.29). Macrofaunal diversity also correlated with both prokaryotic (*p* = 2E-8, ρ = 0.38) and fungal (*p* = 2E-6, ρ = 0.32) rarefied richness.

**Figure 3:**
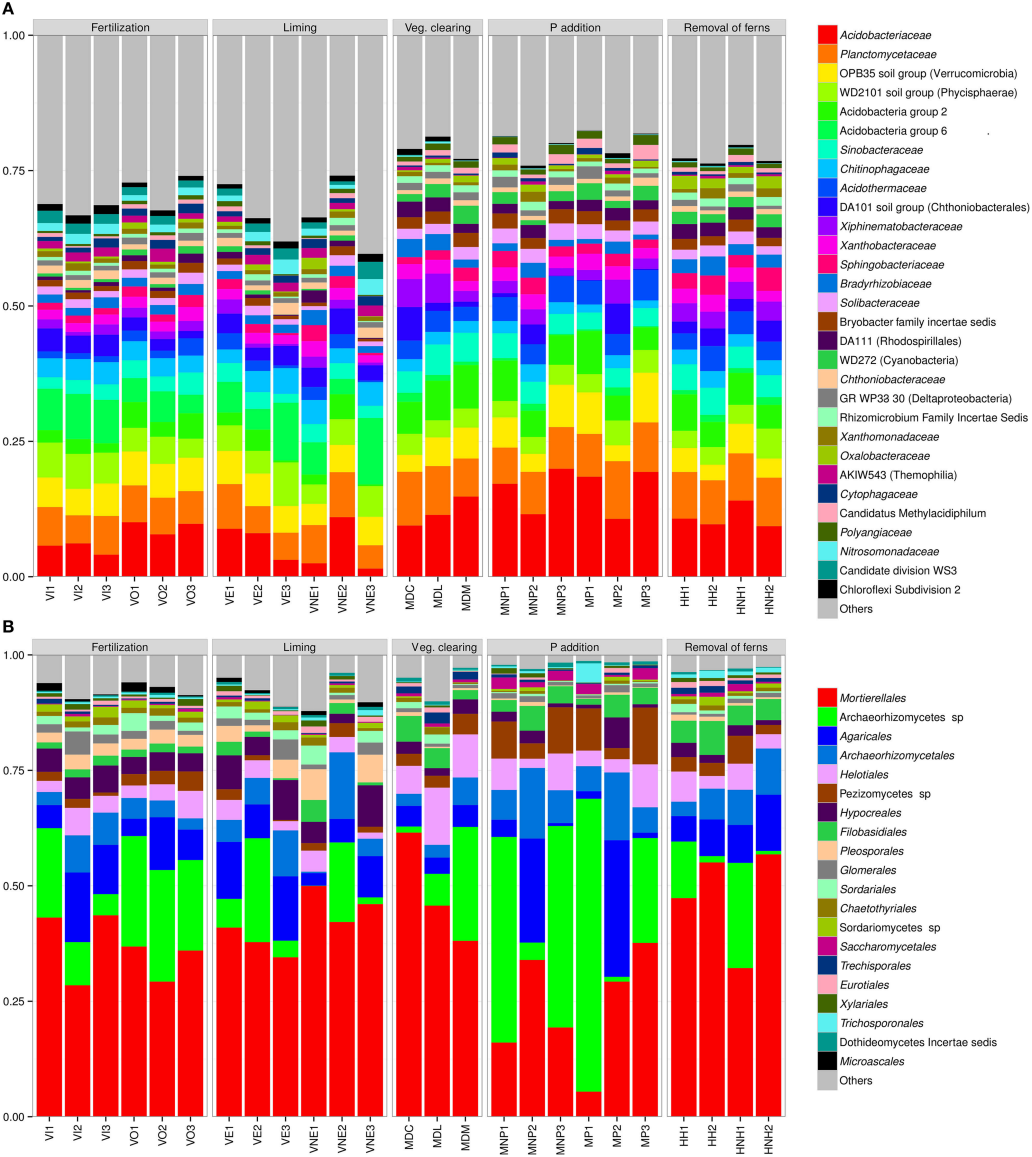
**Barplots representing the distribution of the 30 most abundant prokaryotic taxa (A; family level or below) and the 20 most abundant fungal taxa (B; order level or below)**.

In total 88% of 16S rRNA sequences could be classified taxonomically to family rank and 73% of the ITS sequences to order rank (note: lower ranks were used when taxonomic information at this rank was missing). Prokaryotic communities were relatively even at this level with the top 30 taxa (Figure 3A) together constituting between 60–82% of total abundance in each site-group. Fungal communities appeared more variable and less even at order rank (Figure 3B) with the two most abundant taxa (*Mortierellales* and *Archaeorhizomycetes* sp.) together contributing over half of total abundance. *Acidobacteriaceae* and *Planctomycetaceae*, followed by Verrucomicrobia OPB35 soil group, were the most abundant prokaryotic taxa.

### Influence of habitat types on environmental parameters and community structure

The sites studied were distributed in three characteristically different elevation zones and differed further with regard to bedrock (parent material), apart from land-use and treatments carried out to evaluate the impacts of agricultural practices. As seen in Figure 2, high mountain (*HM*) sites tended to have lower belowground diversity, while valley (*V*) sites were more diverse. Many other environmental parameters also differed significantly between *M* and *V* sites (*M* resulting from merging *LM* and *HM* sites, between which no significant differences were identified; see Figure S1). Pasture production and CO_2_ emission was generally higher in valleys, whereas parameters associated with carbon sequestration (soil organic matter, soil color, and root abundance) were higher in *M* (as expected). The most significant difference was soil pH, with mountain sites being more acid, as expected (pH range 3.7–5.3 for *M* and 5.0–7.8 for *V*). The associated concentration of Al^3+^ cations (hereafter “Al saturation”), known to generally pose a stress to organisms in acid soils, was also higher in *M*.

Microbial diversity in sites with siliceous bedrock did not differ significantly from those with calcareous, or vice versa. However, calcareous sites had more diverse plant communities, lower root abundance, and (as expected) lighter soil color (Figure S2).

Consistent with the differences in diversity and environmental parameters, abundances of the most common taxa appeared to differ most between mountain and valley sites (Figure 3). Of the 609 identified taxa, 530 (87%) were shared between *M* and *V* (detected at least once at both elevation zones) and 64 unique to *V*. Similarly, 73% of OTUs were detected in both *M* and *V*. Most differences between these environments, also for rare taxa, were thus reflected in relative abundance of taxa rather than presence/absence.

**Figure 4:**
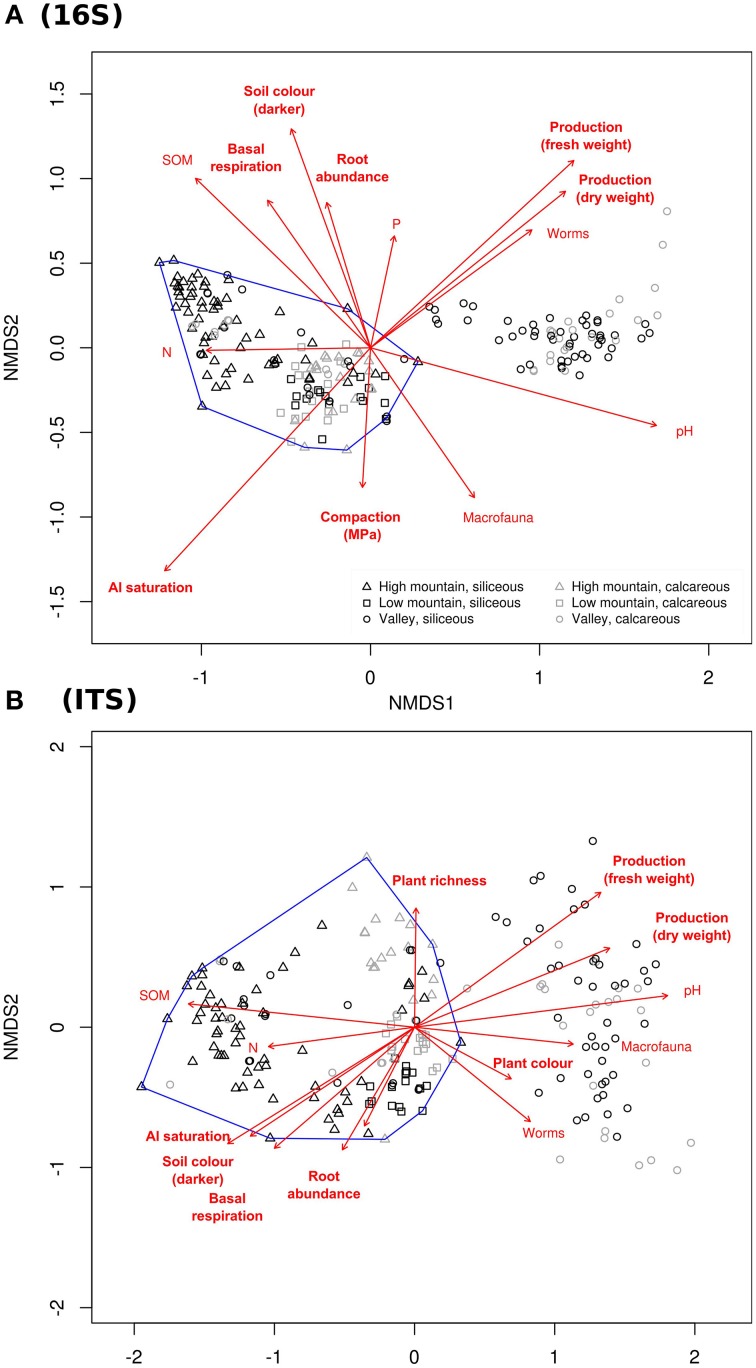
**Non-metric multidimensional scaling (NMDS) based on Bray-Curtis dissimilarities of community composition**. Composition was based on relative OTU abundances from **(A)** prokaryotic 16S and **(B)** fungal ITS amplicon data. Sites are labeled according to legend and the area containing all mountain sites is enclosed by blue lines. Red vectors indicate fitted environmental parameters significantly correlated to NMDS coordinates.

Ordination (NMDS) based on OTU composition of individual samples (Figure 4) also resulted in a clear separation of mountain from most valley sites. Both for 16S rRNA and ITS amplicons, all high- and low mountain sites formed a separate cluster that also contained a minority of the valley sites (Figure 4). These deviating valley sites were distributed equally between relatively diffuse sub-clusters, most *LM* sites and those with calcareous bedrock forming a separate cluster from most *HM* sites. These patterns were verified by ANOSIM (see Table S3).

### Influence of environmental parameters on community structure

**Figure 5:**
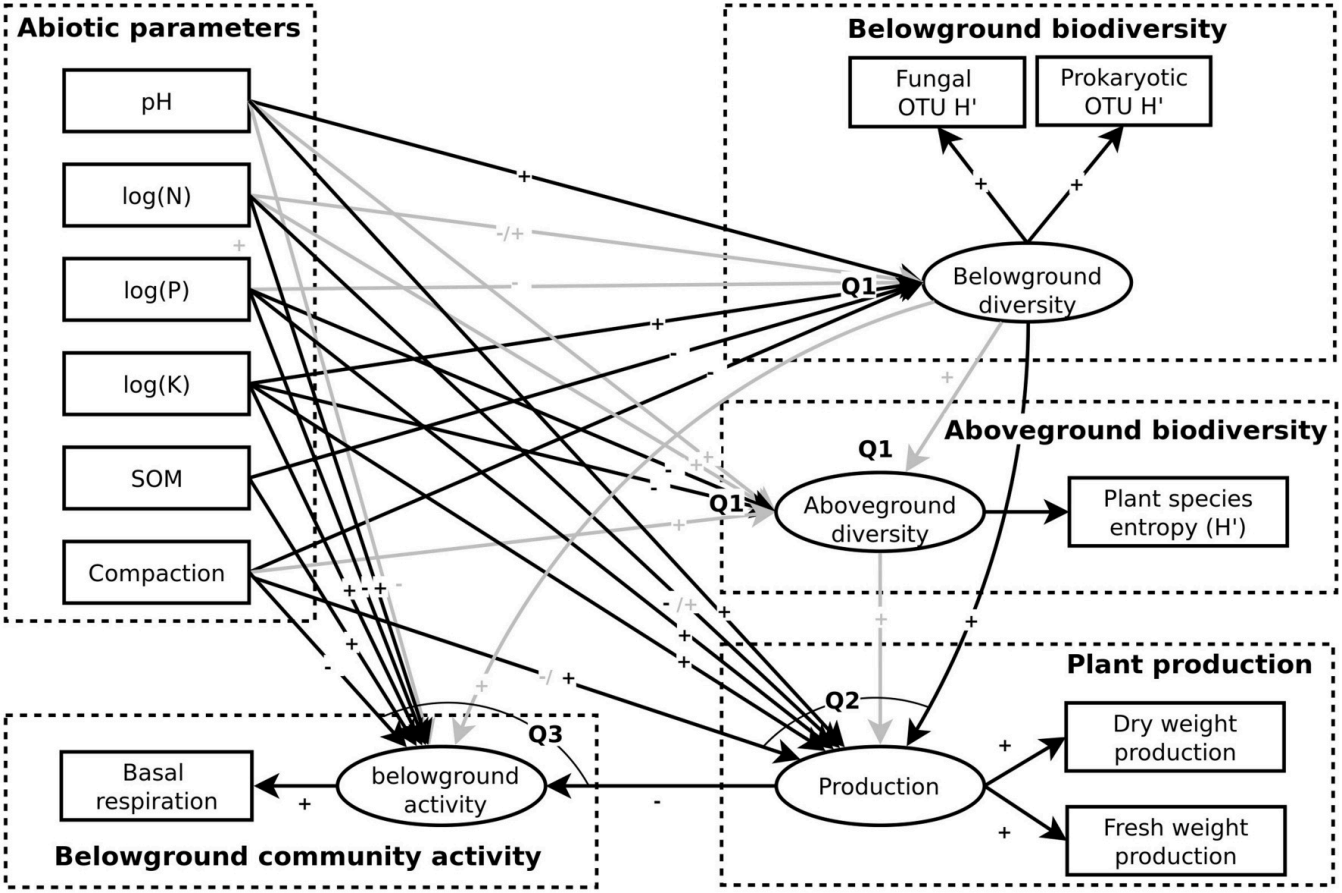
**Structural Equation Model (SEM) derived from the theoretical meta-model of the community (see Figure 1) and corresponding questions targeted**. Latent variables are illustrated as ellipses, exogenous variables are as boxes and theoretical constructs as boxes with dashed lines. Causal links indicated as significant in the total or group-wise (*M* only) best-fitting maximum likelihood solution to the model (see Table S4) are indicated in black while others are indicated as gray. Positive or negative correlations are indicated by ± signs and when differing between the fit for the total dataset and *M* both are indicated.

A Structural Equation Model (SEM) was developed (Figure 5) in order to assess the influence of selected abiotic parameters on belowground community diversity (Q1), the influence of the later and the same parameters on aboveground plant diversity (Q1) and all of the former on above- and below-ground community activity (indicated by plant production and basal respiration, respectively; Q2–3). Fitting this model to the data available (see Table S4 and annotations in Figure 5) indicated that belowground diversity was mainly determined by pH and extractable K concentration, both having a positive influence. Soil organic matter (SOM) and compaction appeared to have a negative influence in *M* sites. K concentration instead appeared to influence plant diversity negatively, together with P. No significant connection between below- and aboveground diversity was supported.

Correlation of environmental parameters to NMDS coordinates (Figure 4) supported SEM results, indicating that pH and SOM also influenced community composition, in addition to overall diversity. For prokaryotes, this was also the case with P and N concentration. pH correlated strongest with both prokaryotic and fungal community structure, followed by Al saturation for prokaryotes and SOM for fungi (see also Table S5).

### Parameters influencing plant production and belowground activity

SEM results (Figure 5 and Table S4) indicated that pasture plant production (Q2) was controlled by pH (with more acid soils having a negative effect), compaction (positive effect in *M*) and nutrient concentrations. The later had contrasting impacts. While total N appeared to negatively influence production overall, it had a weak positive, but non-significant (*p* < 0.1) effect in *M* sites. P and K concentrations instead had consistently positive impact according to the model. NMDS supported the correlation of community structure with plant production (Figure 4).

According to the SEM, belowground activity (Q3), as indicated by basal respiration, appeared negatively influenced by plant production, possibly indicating above- and below-ground competition. Activity was also significantly influenced by compaction; and the nutrients P, N, and K; with the former two having a negative and the later two a positive effect (see Figure 5 and Table S4). Consistent with these results, plant production and basal respiration showed opposite correlations with the NMDS axes representing community structure (Figure 4).

Induced respiration is an alternative indicator of belowground activity, indicating its potential rather than current activity. Although, highly correlated to each other, induced respiration was positively correlated to pH, whereas basal respiration showed the opposite trend (i.e., higher in acid soils, *p* < 2E-16). An alternative version of the SEM using induced respiration supported this positive influence of pH (*p* < 1E-3) and also indicated a positive influence of belowground diversity. The later was also supported by earthworm abundance, as yet another alternative indicator of activity, which strongly correlated with rarefied richness and other diversity estimates based on fungal and prokaryotic amplicon data (*p* < 3E-7; ρ = 0.4, 0.3 respectively).

### Influence of environmental parameters on individual taxa

The SEM and NMDS analyses described above could only take into account correlations to overall diversity and community structure, respectively. In order to study impacts on individual taxa of parameters determined to influence or be influenced by the belowground community, we carried out individual rank correlations to relative taxon abundances. This resulted in 38 significantly affected taxa (consistently in both *M* and *V;* Table S6). Consistent with pH being a strong influence, 33 of these appeared influenced by pH or the associated Al saturation. Another, 10 taxa were significantly correlated to pH in both *M* and *V*, although inconsistently, being more abundant in acid valley sites, but also in basic mountain sites, indicating an intermediate optimum around pH 5 (Table S6 and Figure S5).

In addition to pH or Al saturation, *Archaeorhizomycetales* (ascomycete fungi) correlated positively to plant richness, and *Streptomycetaceae* (Actinobacteria) negatively with basal respiration and N content. *Rhytismatales* (ascomycete fungi with several examples of plant pathogens) and Armatimonadetes Group 1 (previously “OP10”) correlated negatively with plant production, in contrast to total microbial diversity. Although K, SOM, and compaction influenced belowground diversity according to the SEM (Figure 5), and composition according to NMDS (Figure 4), no effect of these parameters on individual taxon abundances could be determined.

### Co-occurrence of taxa

In addition to the influence of environmental parameters, taxa in the soil ecosystem are expected to influence each other through synergistic, trophic or competitive interactions (Figure 1: Q4). This may explain the strong correlation between fungal and prokaryotic diversity observed. A Mantel-test was also used to compare the Bray-Curtis dissimilarity matrices resulting from fungal and prokaryotic OTU distributions, indicating a strong degree of correlation between the community compositions among these two life forms (*p* < 1E-3, *R* = 0.84).

**Figure 6:**
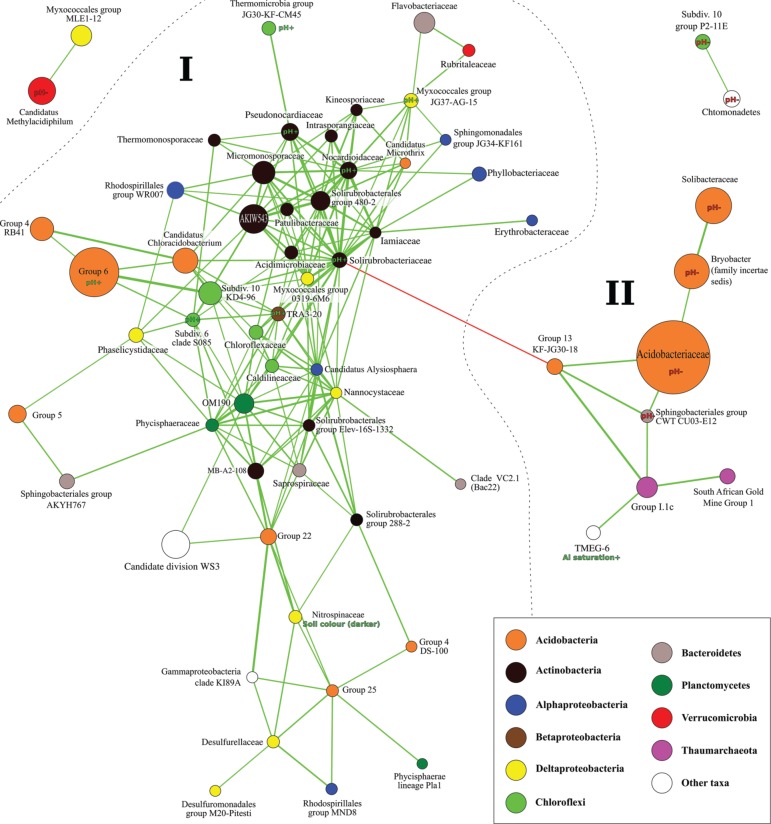
**Kendall rank-correlation network of taxa based on relative abundances across samples**. Taxa are represented by circles and colored according to taxonomic identify (see legend). Circle size is proportional to average abundance across datasets (cut-off for inclusion = 0.01%) and thickness of edges to strength of correlation (cut-off for inclusion: |τ| > 0.8). Significant correlation to environmental parameters is annotated.

To allow for identification of interacting taxa, we used a Kendall rank-correlation network to identify groups of co-occuring taxa (Figure 6). Although included in the analysis, no fungal taxa appeared in the network. One large and relatively tightly coupled module was identified (I), negatively correlated to a smaller module (II). Module II was dominated by the abundant *Acidobacteriaceae*, which along with three other taxa in the module (two acidobacterial), were significantly more common in acid soils (Figure 6, Table S6). Module I, on the other hand was dominated by Acidobacteria Group 6 favored instead in basic soils, as well as eight other taxa. This indicates that the pH of a site may act to favor either one of the two modules, its taxa in turn favored by trophic or synergistic interactions. It is more challenging to predict the nature of such interactions, but among the most connected taxa in Module I were several Actinobacteria, Myxobacteria and the unclassified family of *Candidatus Alysiosphaera*.

### Influence of land-use and experimental treatments

Significant differences associated with the land-use in *V* were identified (Q5). Pastures grazed year-round (*P*) showed lower plant diversity, lower compaction, and higher P and K concentrations, compared to mixed land-use (*X;* winter grazing only) or non-grazed harvested sites (*H;* see Figure S3). The later showed higher Al saturation. Both prokaryotic and fungal community composition differed significantly depending on land-use (Table S3). Out of 10 bacterial taxa with significantly different abundance, eight were most abundant for mixed land-use (Figure S3).

**Figure 7:**
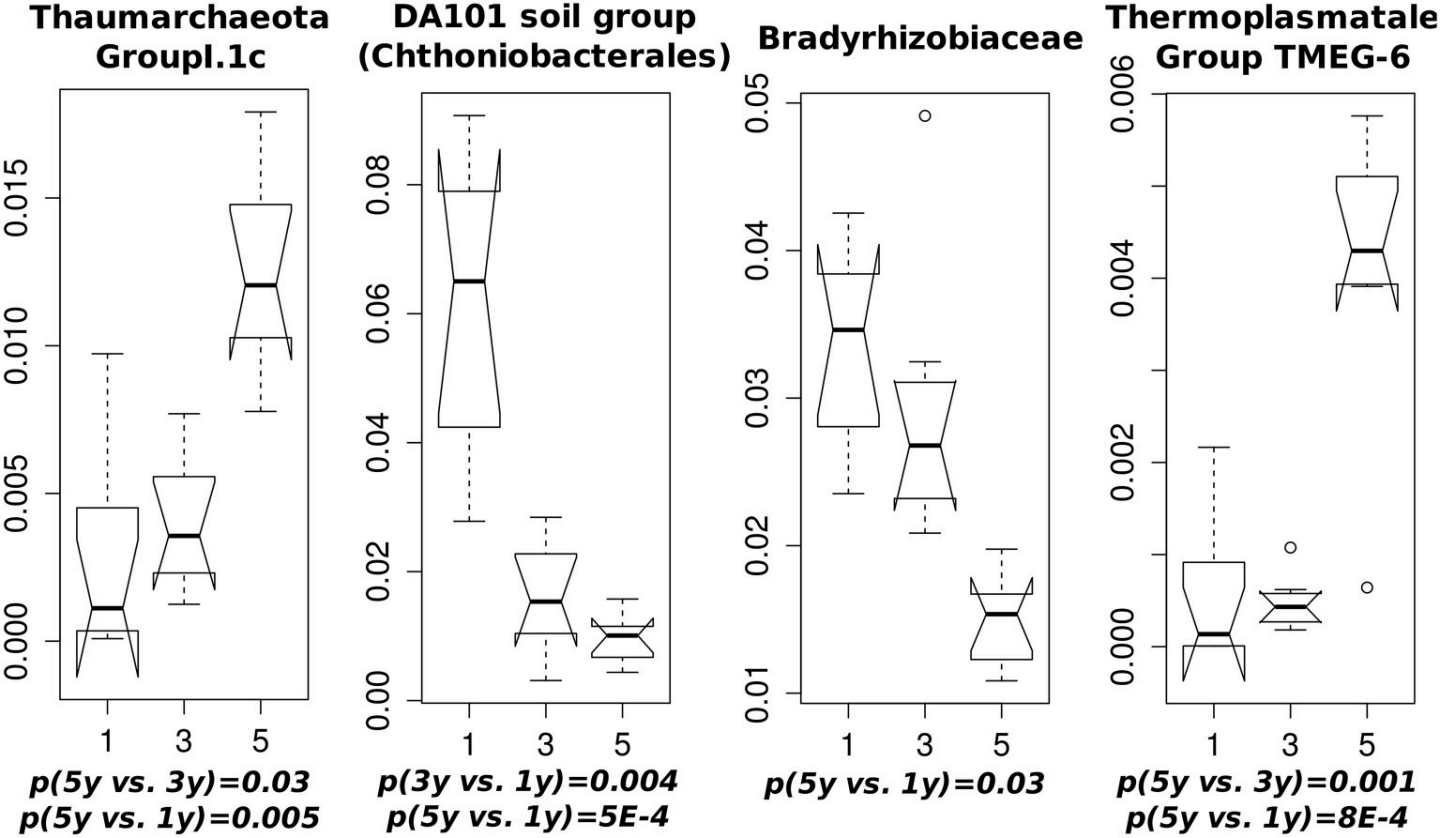
**Taxon abundances showing significant differences in sites cleared from bushes 1, 3 and 5 years prior to first sampling**. Width of notches indicates 95% confidence intervals of median relative abundance. *p*-values determined by group-wise ANOVA (and verified by Tukey's range test) are given below each boxplot.

None of the experimental treatments showed significant effects on environmental parameters. However community composition differed significantly between the sites cleared from bushes 1, 3, or 5 years prior to first sampling (Figure 7). Other treatments did not show significant effects, although in sites with calcareous bedrock, communities subjected to herbicide treatment differed significantly from those cleared mechanically from ferns (Table S3). No effect on individual taxa could be identified.

In addition to the factors investigated, several environmental parameters also varied significantly between the two sampling years (see Figure S4). While respiration decreased from 2013 to 2014, 16S rRNA richness increased along with pasture production. This may have been caused by local weather conditions or possibly sampling bias. However, since the same treatments and sites were studied both years, it was not considered a confounding factor.

## Discussion

### Influence of abiotic parameters on below- and above-ground communities (Q1)

This study represents one of the most ambitious simultaneous surveys targeting both (1) important physiochemical and biological properties and (2) prokaryotic and fungal soil community diversity, using a contemporary culture-independent approach (16S rRNA and ITS amplicon sequencing). Overall, pH showed the strongest influence on composition and diversity of prokaryotic and fungal soil communities. This is consistent with previous studies, including several utilizing current-generation amplicon sequencing to target fungal or bacterial communities (Rousk et al., 2010; Shange et al., 2012; Tripathi et al., 2012; Zhalnina et al., 2014). Our results also support the notion that acid soils tend to have lower diversity. However, the same number of specifically acidophilic and alkaliphilic taxa were identified, as well as several appearing to prefer intermediate pH ranges (Table S6 and Figure S5).

Consistently with Rousk et al. (2010), Acidobacteria both preferring acid soils (including subgroups 1, 2, and 3) as well as basic soils (subgroup 6) were identified. Similarly, groups of Bacteroidetes, Actinobacteria, Chloroflexi, Verrucomicrobia, Proteobacteria, and Armatimonadetes beyond phylum rank, which is unfortunately not reported in many studies. Several taxa preferring higher pH occurred in the largest cluster of co-occuring taxa identified, including the highly connected Actinobacteria *Solirubrobacteriaceae* and *Nocardioidaceae*. In contrast, taxa preferring lower pH were common in three smaller clusters (Figure 6). This indicates the presence of a more complex interaction structure in neutral or basic soils, and can possibly help to explain how soil pH structures microbial diversity in the communities investigated.

Regarding plant diversity, calcareous semi-natural grasslands are among the most species-rich communities in temperate Europe (Kull and Zobel, 1991). Consistently, sites with siliceous bedrock showed relatively lower plant richness (Figure S2). However, no correlation between plant richness and pH could be identified, whereas earlier studies have shown lower plant diversity in acid soils (Zhalnina et al., 2014). In spite of the importance of plant-microbial (above- and below-ground) interactions (van der Heijden et al., 2008), plant and microbial diversities did not appear related, similar to findings of Zhalnina et al. (2014) and Prober et al. (2015). Plant richness was also negatively related to phosphorus and potassium content in valleys, where many sites contained concentrations above those optimal for biodiversity (approximately 50 and 200 mg kg^−1^ soil, respectively according to Janssens et al., 1998). Curiously, potassium appeared to have the opposite effect on microbial diversity.

As opposed to alpha diversity, plant richness correlated positively with the abundance of the recently discovered fungal taxon *Archaeorhizomycetes* sp. Its ecological role is unclear, but species are thought to interact with plants through different strategies ranging from endophytic to free-living saprophytic (Rosling et al., 2013). It is interesting to note that this taxon was found in relatively high abundance throughout our samples (see Figure 3B), especially in light of the recent debate concerning its global abundance (Schadt and Rosling, 2015; Tedersoo et al., 2015). Here, the ITS1 region was targeted using primers ITS1F and ITS2 (White et al., 1990; Gardes and Bruns, 1993). The resulting relative abundance of 20% is over tenfold as high as suggested by a global survey of Tedersoo et al. (2014) targeting the ITS2 region, using a mix of ITS3 primers and a novel version of primer ITS4, the later thought to be biased against *Archaeorhizomycetes* (Schadt and Rosling, 2015). However, it is not clear whether the primers used here over-estimates the abundance of *Archaeorhizomycetes*, whether the ITS4 primers used by Tedersoo et al. (2014) underestimates it, or both. Clearly further studies are necessary to resolve this issue.

### Factors determining pasture production (Q2) and belowground activity (Q3)

Plant production appeared positively correlated with pH. As expected, it was also higher in valleys, as was pH. Plant production also appeared positively influenced by potassium and phosphorus content, as opposed to their negative effects on plant diversity, indicating that these nutrients were rate-limiting for production, although present above the optimal values for plant diversity in many sites. This would agree with the well-known theory of a hump-backed rather than linear relationship between plant biodiversity and productivity (Grime, 1973). Belowground diversity, on the other hand, showed a significantly positive influence on plant production. This result is expected, considering the different beneficial above- and belowground interactions known, including directly via root-associated microbes, and indirectly via free-living microbes contributing to nutrient supply (Barrios, 2007; van der Heijden et al., 2008).

Belowground activity as indicated by basal or induced respiration appeared controlled by similar factors as belowground diversity. However, available phosphorus content (Olsen P) appeared to be a negative influence, as well as plant production. This may indicate relatively higher belowground activity of mycorrhizal fungi or P-solubilising bacteria in less productive sites limited by this nutrient. Similarly, total nitrogen on activity, as opposed to plant production, may indicate active denitrification. However, nitrogen or nitrous oxide emission was not measured in order to verify this. Further, these speculations depend on the SEM approach used here (Figure 5). While useful as a statistical and interpretative tool, it also represents a simplification of true ecosystem processes, which also include causal feedbacks. These were not possible to model and fit with the data considered here.

An alternative interpretation explaining the negative correlation between production and belowground activity is that highly productive sites were exposed to more intensive grazing, thus limiting litter decomposition. Grazing pressure was not measured or included in the model. To do so is challenging, as animals were free to move between mountain sites. However, grazing would also explain why production appeared positively influenced by soil compaction, which otherwise is counter-intuitive.

### Interactions between microbial taxa (Q4)

Prokaryotic and fungal alpha diversity was strikingly correlated across investigated sites (Figure 2). Likely, this can be partially explained by similar influences from parameters such as pH, macrofaunal diversity and SOM on fungal and prokaryotic diversity (Figures 4, 5). However, the strength of the correlation between rarefied fungal and bacterial richness suggests a more direct relationship. We suggest that interactions are likely responsible for this relationship, since fungal and prokaryotic interactions typically play vital roles in many ecosystems including soil and can lead to the formation of interdependent fungal-bacterial consortia, with emergent properties (Tarkka et al., 2009; Frey-Klett et al., 2011).

It is likely that interactions also explain the similar influences on fungal and prokaryotic composition revealed by ordination and SEM analyses (Figures 4, 5), consistent with earlier studies of such influences (van der Heijden et al., 2008; Burke et al., 2012). Although, no specific fungal-prokaryotic interactions were identified using taxon correlation network analysis (Figure 6), it is possible that they were obscured by the relatively higher heterogeneity in ITS amplicon data compared to 16S rRNA. We expect this to result from the small amount of soil from which DNA was extracted (0.25 g). It is expected that larger and elongated structures such as fungal hyphae are more patchily distributed at this scale with a more heterogeneous distribution compared to smaller bacterial cells.

Both fungal and prokaryotic richness also correlated with macrofaunal diversity, which can likely be explained by the presence of a diverse range of microbial endo- and exo-symbionts of macrofauna, as well as trophic interactions between the soil micro- and macrofauna. Consistent with this, abundance of earthworms also correlated with prokaryotic richness.

Few studies have previously compared fungal and prokaryotic diversity estimates using high-throughput culture-independent sequencing across a similarly large number of samples as here. Prober et al. (2015), however, utilized a similar approach as ours for this purpose, studying 145 geographically dispersed grassland sites from four continents. As opposed to this study, no significant correlation between fungal and prokaryotic richness was identified. If our hypothesis regarding interactions structuring the correlation between fungal and prokaryotic communities is correct, then it is possible that the failure to detect this relationship by Prober et al. (2015) was caused by the larger geographical and environmental differences between sites, implying a smaller overlap of fungal and prokaryotic strains. In other words, the trend observed here may only exist at local or regional scales, where the presence or absence of particular prokaryotic taxa often implicate presence or absence of corresponding fungal taxa. It does not necessarily imply that the diversity of fungi and bacteria correspond to each other in a similar way over larger interregional or global scales.

### Influence of land-use and management practices (Q5)

Contrary to expectations, no experimental treatments resulted in significant changes in measured parameters like pH, pasture production or mineral levels. In the case of liming, this may be explained by the fact that sampling was carried out relatively soon after application (approximately 3 months) and the moderate amounts of calcium hydroxide applied. Organic phosphate fertilization in mountain sites was carried out in relatively low doses, due to environmental regulations due to the protection status of Gorbeia Natural Park. Further, negative control was not possible for fertilization in valley sites where either inorganic and organic fertilizer was applied, in similar dosage. Previous studies have also shown that impact of fertilization regimes on plant and microbial diversity may be complex, mediated by changes in pH and require long-term experiments (Tan et al., 2013; Zhalnina et al., 2014; Hartmann et al., 2015).

As opposed to other treatments, clearing of bushes appeared to significantly change individual taxon abundances (Figure 7). The most abundant of these, Thaumarchaeota Group I.1c appeared to prefer more densely vegetated sites compared to recent clearings, along with another archaeon, Themoplasmatales group TMEG-6. This group has previously been encountered in high abundance in acidic forest soils, but opposed to other Thaumarchaeota it appears to lack the ability for ammonia-oxidation, suggesting instead a heterotrophic lifestyle (Weber et al., 2015). Little is known about TMEG-6 and members of this group were identified from environments as divergent as petroleum contaminated soil (Kasai et al., 2005) and floating filaments in extremely acidic water (García-Moyano et al., 2007). DA101 Soil Group of Chtoniobacterales and *Bradyrhizobiaceae* instead appeared favored by recent clearing. The former shows similarity to slow-growing chemoheterotrophs found in bulk soils and may be involved in litter degradation (Yarwood et al., 2013). They are likely nitrogen-fixing root-nodule associated rhizobia favored by the vegetation shift from bushes to legumes, since the overwhelming majority of sequences from this family were classified to genus *Bradyrhizobium*.

Pastured valley soils appeared to be less compacted compared to other land-uses (mixed or harvested). This counter-intuitive relation was likely an effect of site selection, and cannot be considered representative for true differences between land-use patterns. Interestingly, sites with mixed land-use (grazed during winter only), appeared to have higher abundance of several anaerobic taxa including three groups of Deltaproteobacteria and the *Anaerolineacea* (phylum Chloroflexi).

## Concluding remarks

As noted for the relation between pasture production and belowground activity, a limitation of the SEM used here is that it is unable to include feedback as causal links. An ecosystem service that is challenging to study using this approach is carbon sequestration. As expected, belowground activity appeared to be positively influenced by organic matter content (Figure 5). However, the accumulation of organic matter should also depend on plant production and belowground activity itself. These expected connections could not be included in the model. The negative correlation between organic matter and belowground diversity may also represent a more indirect mechanism in reality, such as increased carbon sequestration in nutrient-poor, acid soils, co-incidentally also having lower biodiversity.

In conclusion, this study highlights the complexity of soil microbial communities and verifies pH as one of the most important factors in structuring soil biodiversity. It also indicates a striking correlation between prokaryotic, fungal, and macrofauna diversity, likely caused by interactions between these life forms. Further studies are needed to better understand such interactions and to target the influence of different pastoral practices on belowground community structure and function, in face of the significant heterogeneity present in soil habitats.

## Funding

This work has been financially supported by the European Union through Soil-Montana (LIFE10NAT/ES/579) project. AL is supported by a personal Marie Curie grant from the European Union (IEF 326582).

## Author contributions

IM, FB, MA, and CG contributed to the conception and design of the study. MA, IMS, FB, and IM carried out field work and laboratory analyses contributing substantially to the acquisition of data analyzed. AL, LE, CG, and IM contributed to the analysis and interpretation of data. AL and LE drafted the manuscript and all remaining authors revised it critically and approved of the final version.

### Conflict of interest statement

The authors declare that the research was conducted in the absence of any commercial or financial relationships that could be construed as a potential conflict of interest.

## Footnotes

^1^ISO (International Organization for Standardization) (2002a). *Soil Quality – Determination of Abundance and Activity of Soil Microflora Using Respiration Curves*. Geneva: ISO 17155:2002.

^2^ISO (International Organization for Standardization) (2002b). *Soil Quality - Laboratory Methods for Determination of Microbial Soil Respiration*. Geneva: ISO 16072:2002.

## Supplementary material

Figure S1 **Parameters showing significant differences between mountain (***M***) and valley (***V***) sites**. Distributions of values measured at each elevation zone are illustrated as box plots. *p*-values determined by group-wise ANOVA (and verified by Tukey's range test) are given below each plot.

Figure S2 **Parameters showing significant differences between sites with siliceous (S) and calcareous (C) bedrock**. Distributions of values are illustrated as box plots. Only differences that could be verified independently in *M* or *V* sites alone are included. *p*-values determined by group-wise ANOVA (and verified by Tukey's range test) are given below each plot (with corresponding values for *M* or *V* subsets in brackets).

Figure S3 **Parameters and taxon abundances showing significant differences with land-use (***P***, pastures grazed year-round; ***X***, winter grazing only; ***H***, non-grazed, harvested)**. Width of notches indicates 95% confidence intervals of the median. *p*-values determined by group-wise ANOVA (and verified by Tukey's range test) are given below each boxplot.

Figure S4 **Parameters showing differences between sampling years (2013 and 2014)**. Significantly different distributions of values measured for each year are illustrated as box plots. *p*-values determined by group-wise ANOVA (and verified by Tukey's range test) are given below each plot.

Figure S5 **Relative abundance as a function of pH for 10 taxa with inconsistent but significant correlation to pH in both ***M*** and ***V*** sites**. Curves fitted using LOWESS (Locally Weighted Scatterplot Smoothing).

Table S1 **Samples with collection date, location, physical parameters measured and other metadata**.

Table S2 **Alpha diversity estimates for all samples**.

Table S3 **Results from Analysis of Similarities (ANOSIM) between communities (Bray-Curtis), asterisks indicating significance strength**.

Table S4 **Best fitting solutions to the structural equation model (Figure 2), asterisks indicating significance strength**.

Table S5 **Parameters correlated to NMDS axes (significant correlations in bold)**.

Table S6 **Taxa with relative abundance significantly correlated to parameters measured in mountain and valley sites**.
