# Supplementary figures and images for: The Community Structures of Prokaryotes and Fungi in Mountain Pasture Soils are Highly Correlated and Primarily Influenced by pH
Source: Front Microbiol. 2015 Nov 27;6:1321. doi: 10.3389/fmicb.2015.01321 (PMC4661322; doi:10.3389/fmicb.2015.01321)

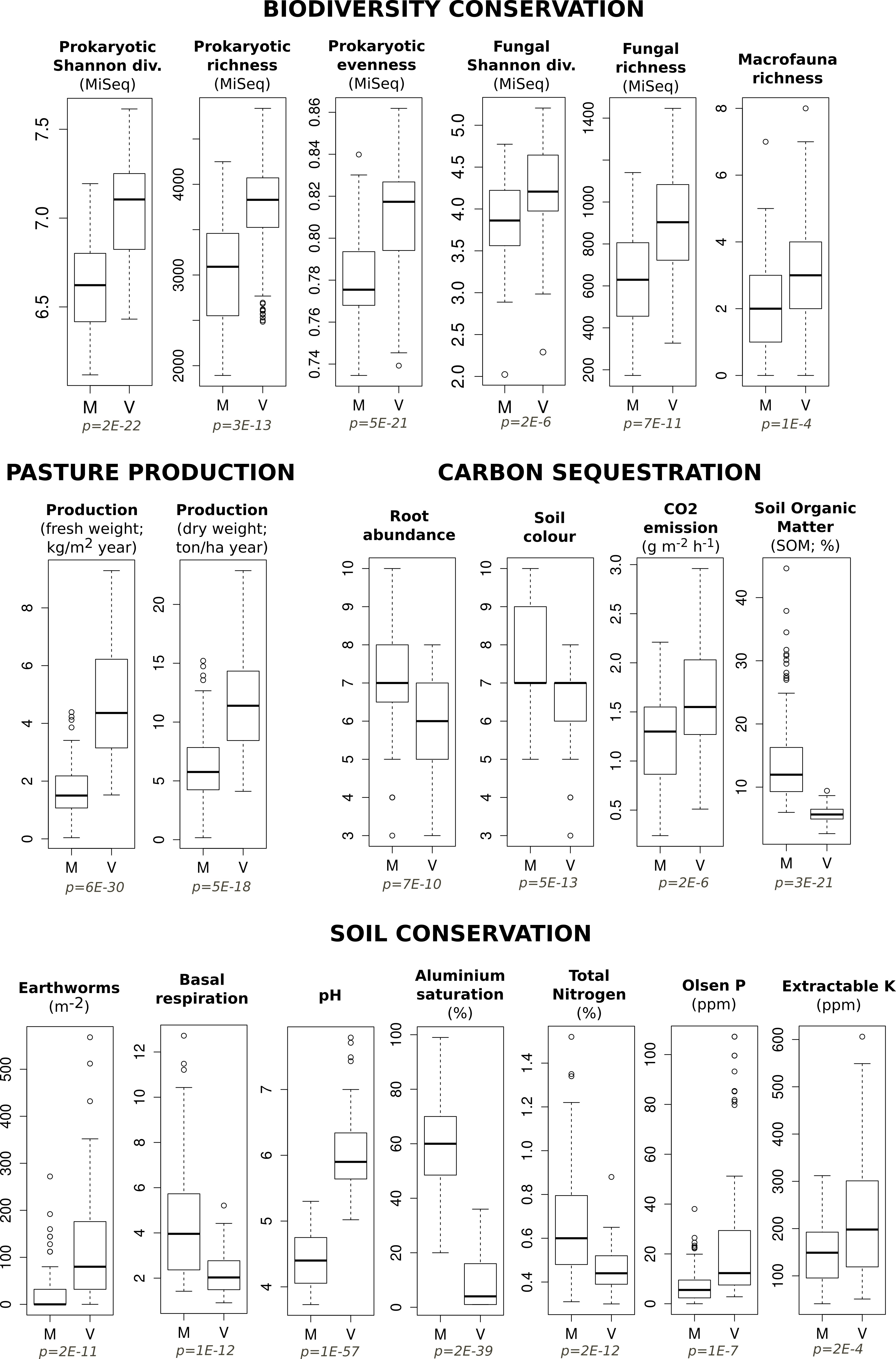

Supplement: Figure S1 — Parameters showing significant differences between mountain (M) and valley (V) sites. Distributions of values measured at each elevation zone are illustrated as box plots. p-values determined by group-wise ANOVA (and verified by Tukey's range test) are given below each plot. [file Image1.JPEG]

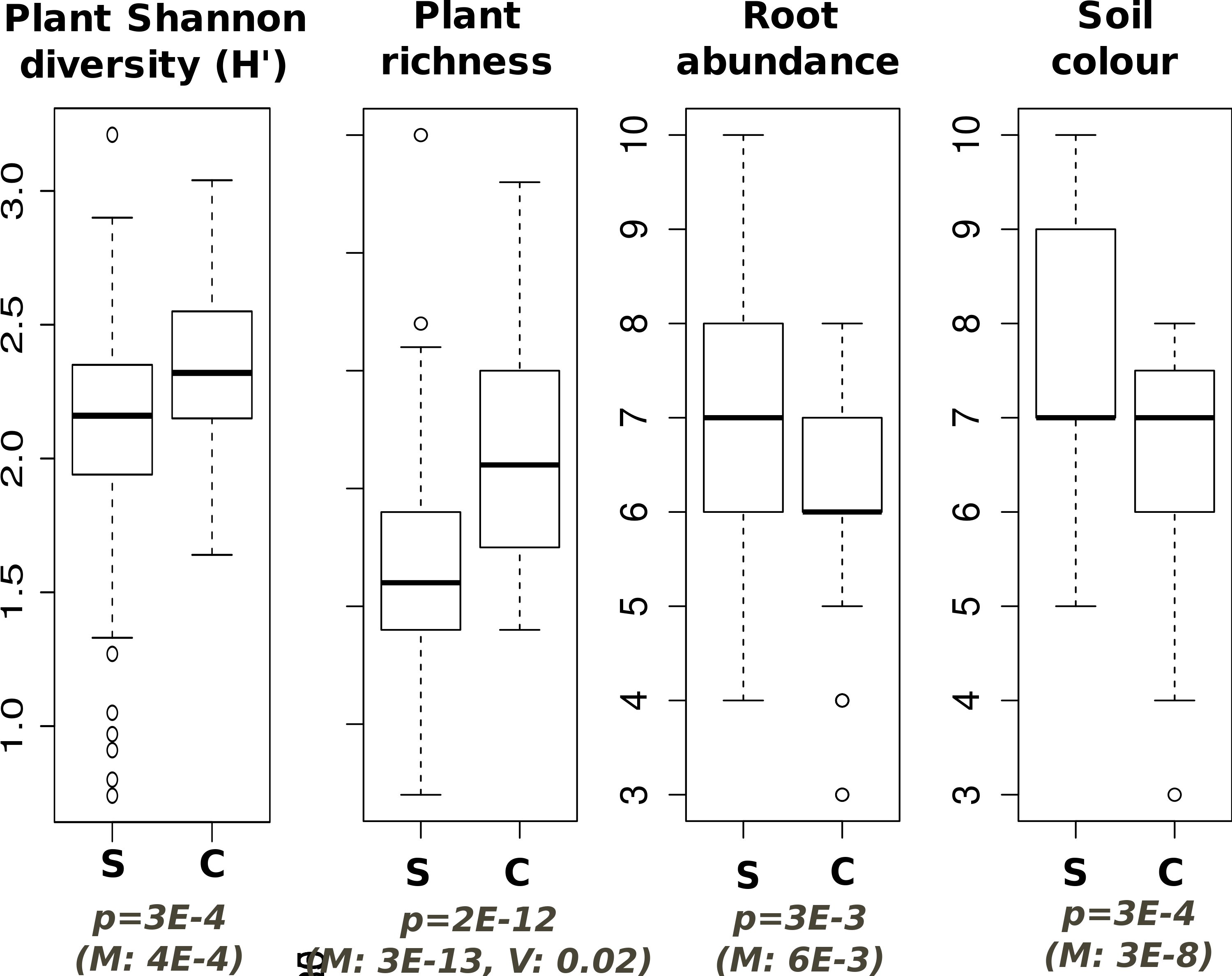

Supplement: Figure S2 — Parameters showing significant differences between sites with siliceous (S) and calcareous (C) bedrock. Distributions of values are illustrated as box plots. Only differences that could be verified independently in M or V sites alone are included. p-values determined by group-wise ANOVA (and verified by Tukey's range test) are given below each plot (with corresponding values for M or V subsets in brackets). [file Image2.JPEG]

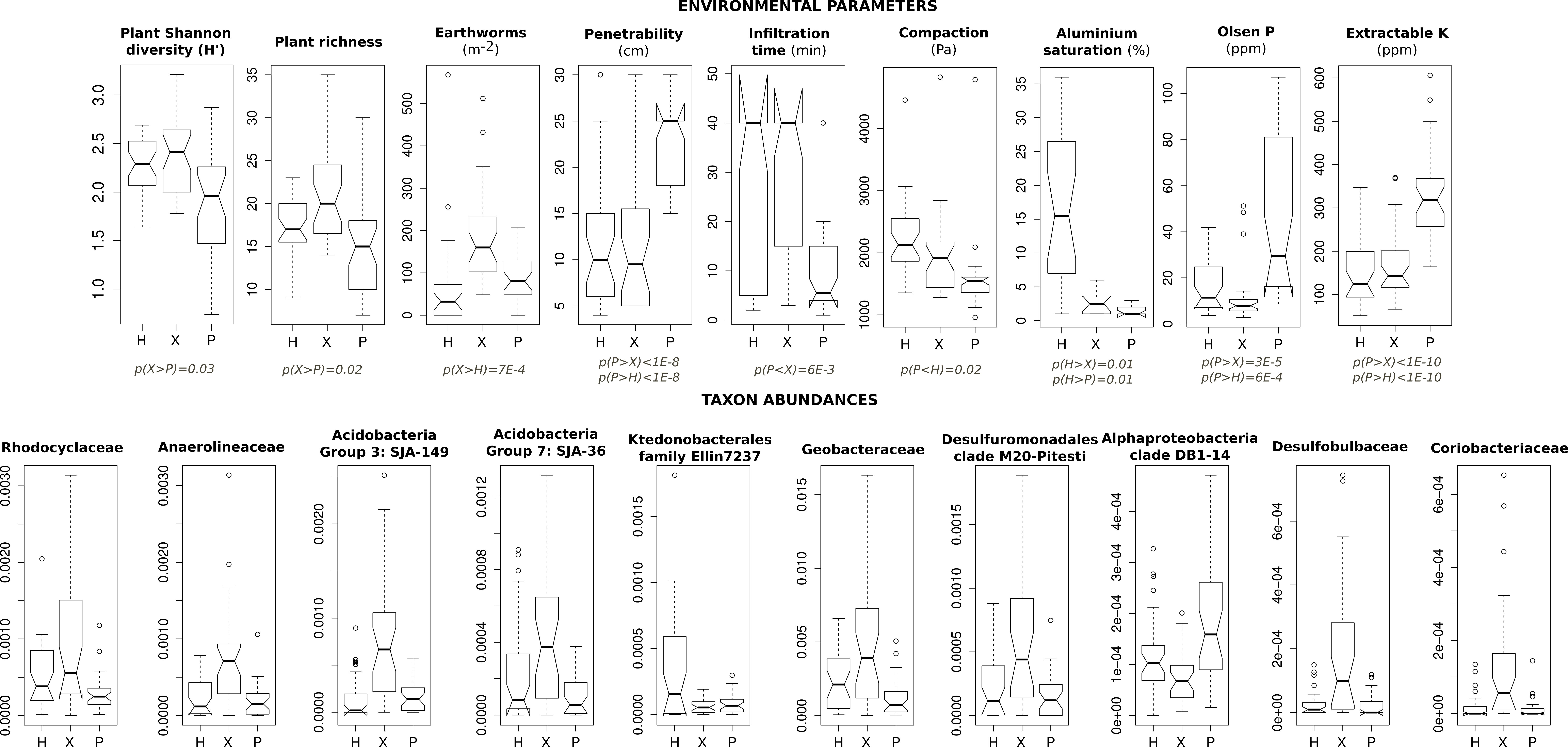

Supplement: Figure S3 — Parameters and taxon abundances showing significant differences with land-use (P, pastures grazed year-round; X, winter grazing only; H, non-grazed, harvested). Width of notches indicates 95% confidence intervals of the median. p-values determined by group-wise ANOVA (and verified by Tukey's range test) are given below each boxplot. [file Image3.JPEG]

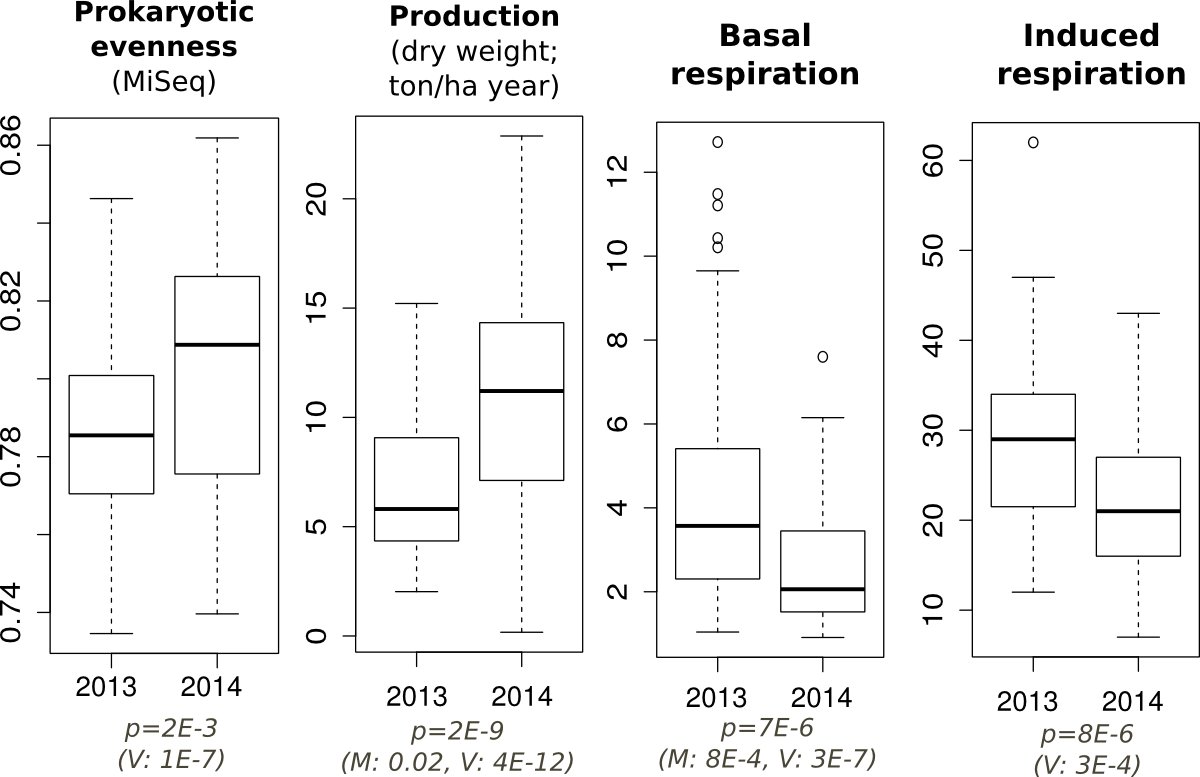

Supplement: Figure S4 — Parameters showing differences between sampling years (2013 and 2014). Significantly different distributions of values measured for each year are illustrated as box plots. p-values determined by group-wise ANOVA (and verified by Tukey's range test) are given below each plot. [file Image4.JPEG]

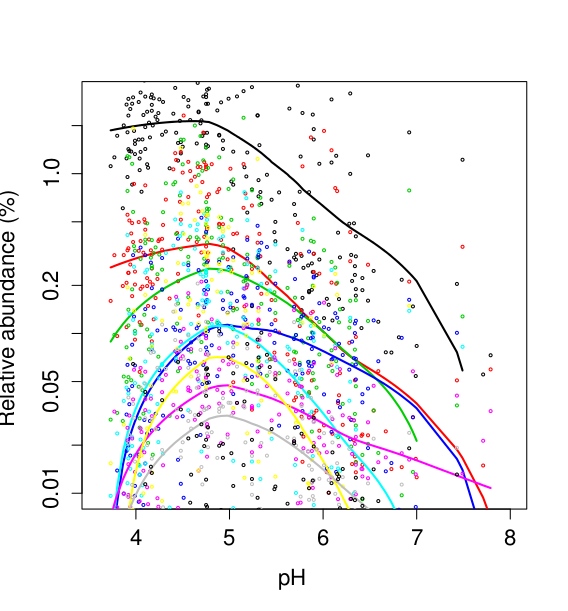

Supplement: Figure S5 — Relative abundance as a function of pH for 10 taxa with inconsistent but significant correlation to pH in both M and V sites. Curves fitted using LOWESS (Locally Weighted Scatterplot Smoothing). [file Image5.JPEG]
