# Supplementary material for: The Community Structures of Prokaryotes and Fungi in Mountain Pasture Soils are Highly Correlated and Primarily Influenced by pH
Source: Front Microbiol. 2015 Nov 27;6:1321. doi: 10.3389/fmicb.2015.01321 (PMC4661322; doi:10.3389/fmicb.2015.01321)
Supplement: Table S3 — Results from Analysis of Similarities (ANOSIM) between communities (Bray-Curtis), asterisks indicating significance strength. [file Table3.DOC]

**Table S3 – Results from Analysis of Similarities (ANOSIM) between communities (Bray-Curtis), asterisks indicating significance strength**.

| **Factor** | **H_0_** | **R (16S)** | **R (ITS)** |
| --- | --- | --- | --- |
| Elevation zone | *HM = LM* | 0.23^***^ | 0.19^***^ |
| Elevation zone | *M = V* | 0.48^***^ | 0.42^***^ |
| Bedrock, all samples | Calcareous = Siliceous | 0.02 | 0.04^*^ |
| Bedrock in M | “ | 0.29^***^ | 0.37^***^ |
| Bedrock in V | “ | 0.06 | 0.39^***^ |
| Plant strata in M | 1S = 2S = 3S | 0.00 | 0.07^*^ |
| Land-use in V | *P = X = H* | 0.08^**^ | 0.09^**^ |
| Time after bush-clearing | MDC = MDL = MDM | 0.46^***^ | 0.52^***^ |
| Fern clearing (herbicide vs. mechanical), calc. bedrock | HH2 = HNH2 | 0.33^*^ | 0.37^*^ |
| Year effect | 2013 = 2014 | 0.11^***^ | 0.10^***^ |
