# Supplementary material for: The Community Structures of Prokaryotes and Fungi in Mountain Pasture Soils are Highly Correlated and Primarily Influenced by pH
Source: Front Microbiol. 2015 Nov 27;6:1321. doi: 10.3389/fmicb.2015.01321 (PMC4661322; doi:10.3389/fmicb.2015.01321)
Supplement: Table S4 — Best fitting solutions to the structural equation model (Figure 2), asterisks indicating significance strength. [file Table4.DOC]

**Table S4 – Best fitting solutions to the structural equation model (Fig. 2), asterisks indicating significance strength.**

| **Response variable** | **Explanatory var.** | ***M+V*** | ***M* only** | |
| --- | --- | --- | --- | --- |
| ***Latent variables:*** |  |  |  |  |
| Belowground diversity | Prokaryotic H' (SSU) | 0.24*** | 0.12*** |  |
|  | Fungal H' (ITS) | 0.25*** | 0.29*** |  |
| Plant production | Dryweight prod. | 2.6*** | 1.8*** |  |
|  | Fresh weight prod. | 1.3*** | 1.2*** |  |
| Aboveground diversity | Vegetation H' | 0.33*** | 0.21*** |  |
| Belowground activity | Basal respiration | 1.3*** | 1.5*** |  |
| ***Regressions:*** |  |  |  |  |
| Belowground diversity | pH | 0.58*** | 1.5** |  |
|  | log(N) | -0.48 | 1.22· |  |
|  | log(P) | -0.03 | -0.18· |  |
|  | log(K) | 0.38* | 0.87** |  |
|  | Compaction | 0.01 | -0.44** |  |
|  | SOM | -0.04 | -0.13*** |  |
| Aboveground diversity | pH | 0.05 | 0.52 |  |
|  | log(N) | 0.37 | 0.39 |  |
|  | log(P) | -0.14* | 0.04 |  |
|  | log(K) | -0.66*** | -0.01 |  |
|  | Compaction | 0.13· | 0.15 |  |
|  | Belowground div. | 0.13· | 0.02 |  |
| Plant production | pH | 0.40** | 0.04 |  |
|  | log(N) | -1.0*** | 0.53· |  |
|  | log(P) | 0.24*** | 0.12* |  |
|  | log(K) | 0.62*** | 0.09 |  |
|  | Compaction | -0.11 | 0.25** |  |
|  | Belowground div. | 0.18* | 0.09 |  |
|  | Aboveground div. | 0.12 | 0.02 |  |
| Belowground activity | pH | -0.23· | -0.69· |  |
|  | log(N) | 1.6*** | 0.75 |  |
|  | log(P) | -0.13* | -0.02 |  |
|  | log(K) | 0.62*** | 1.3*** |  |
|  | Compaction | -0.15* | -0.35** |  |
|  | Belowground div. | 0.11 | 0.05 |  |
|  | Plant production | -0.21** | -0.07 |  |
|  | SOM | 0.09*** | 0.05· |  |
