# Supplementary material for: The Community Structures of Prokaryotes and Fungi in Mountain Pasture Soils are Highly Correlated and Primarily Influenced by pH
Source: Front Microbiol. 2015 Nov 27;6:1321. doi: 10.3389/fmicb.2015.01321 (PMC4661322; doi:10.3389/fmicb.2015.01321)
Supplement: Supplementary file 12 [file Presentation1.PDF]

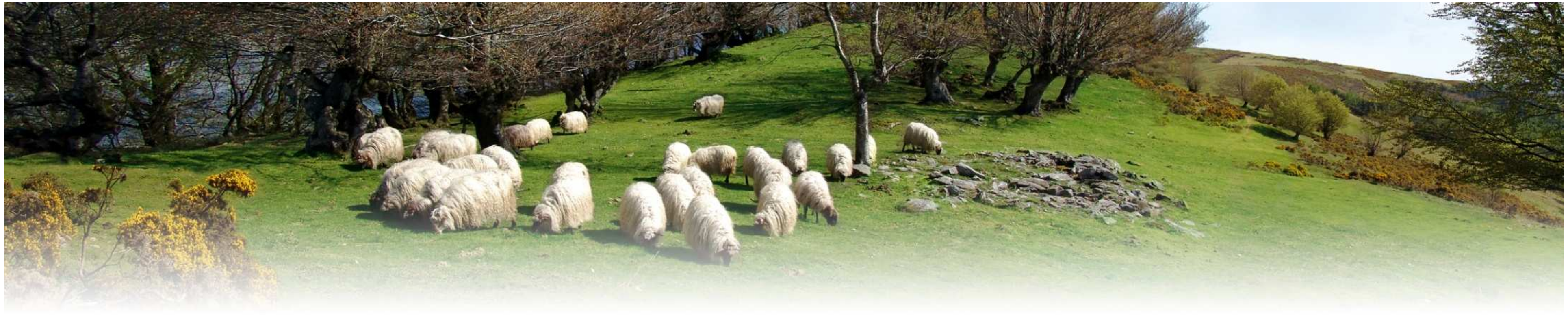

Agroecosystem Health Cards

- GRASSLANDS -

Dr. IKER MIJANGOS (imijangos@neiker.net)
Dr. CARLOS GARBISU (cgarbisu@neiker.net)

AGROECOSYSTEM HEALTH CARDS

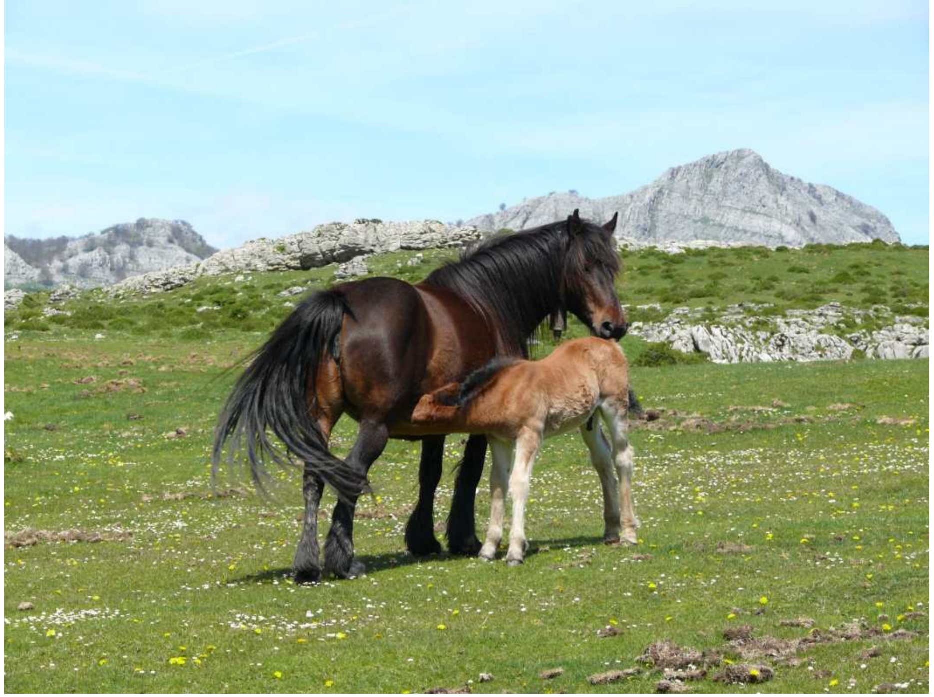

Agroecosystem Health Cards (AHCs) are handbooks that provide straightforward, practical explanations on how to assess the health of agroecosystems. AHCs allow the quantitative assessment of the impact of human activities (*e.g.*, agricultural practices) on agroecosystem health.

AHCs include a variety of indicators of agroecosystem health and provide practical information on how to measure them, together with qualitative and quantitative criteria for data interpretation.

In our AHCs, indicators are grouped into a variety of ecosystem services to facilitate interpretation by decision-takers and, above all, to provide stability to long-term monitoring programs against changes in methods, parameters, etc.

Recommendations on good agricultural practices to improve agroecosystem health are included.

AGROECOSYSTEM HEALTH CARD FOR GRASSLAND ECOSYSTEMS

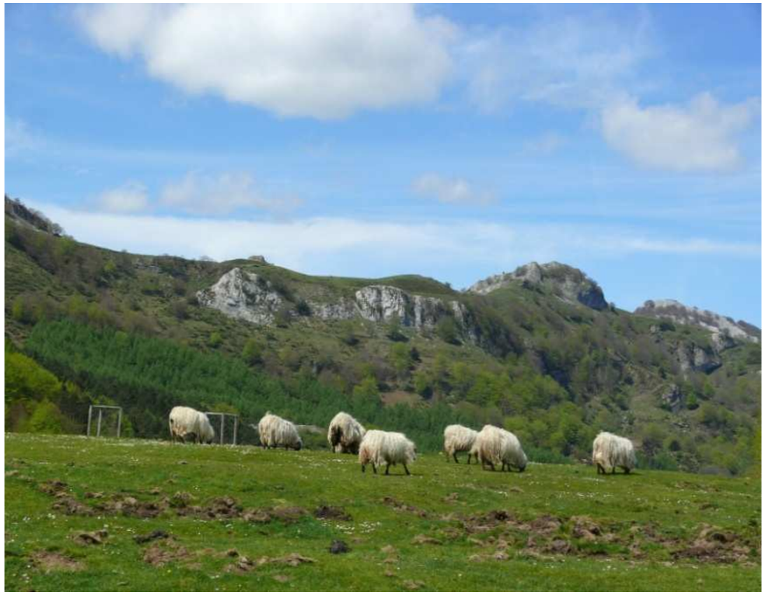

This AHC was **specifically designed for grassland ecosystems. Then, it cannot be used for other types of ecosystems.**

It was developed by NEIKER - The Basque Institute of Agricultural Research and Development, thanks to financial support from the European Union (LIFE+ Programme - LIFE10NAT/ES/579 SOILMONTANA project), in collaboration with:

- Zeanuri and Orozko Stockbreeders Associations
- LORRA Agro-Stockbreeding Cooperative
- Biscay County Council
- Basque Government

The main objective of the LIFE-SOILMONTANA project was to (i) assess the impact of agricultural practices frequently carried out in grazing areas of the Gorbeia National Park (northern Spain) and its surroundings, and (ii) encourage the use of more sustainable agricultural practices from both a socioeconomic (exploitation of livestock resources) and environmental (preserving biodiversity and combating climate change) point of view.

WHO CAN USE OUR AGROECOSYSTEM HEALTH CARDS?

Anyone can use them. For this purpose, AHCs include a number of **BASIC INDICATORS** that can be measured and interpreted without any special training or qualification.

How can we measure these basic indicators? Simply by reading the AHC and using homemade tools and equipment. For this **basic diagnosis** of agroecosystem health, information is provided in the AHCs for non-experts to directly measure *in situ* some indicators, in order to diagnose the health of a specific agroecosystem at a basic level.

For a more comprehensive assessment of agroecosystem health (*i.e.*, **advanced diagnosis**), a number of **ADVANCED INDICATORS**, which require more sophisticated equipment and prior training and qualification, have to be measured.

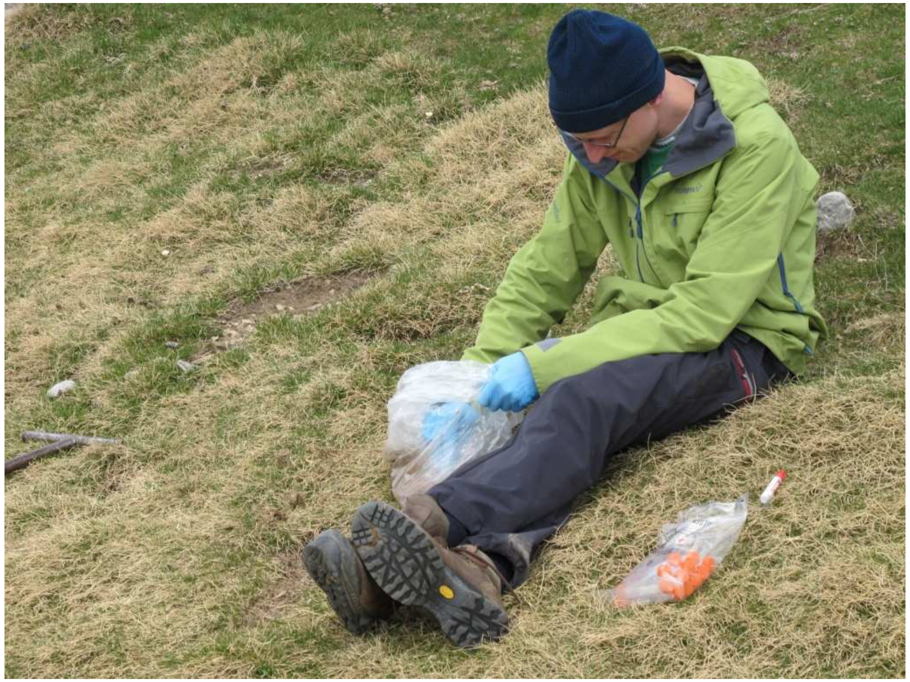

DETERMINATION OF AGROECOSYSTEM HEALTH

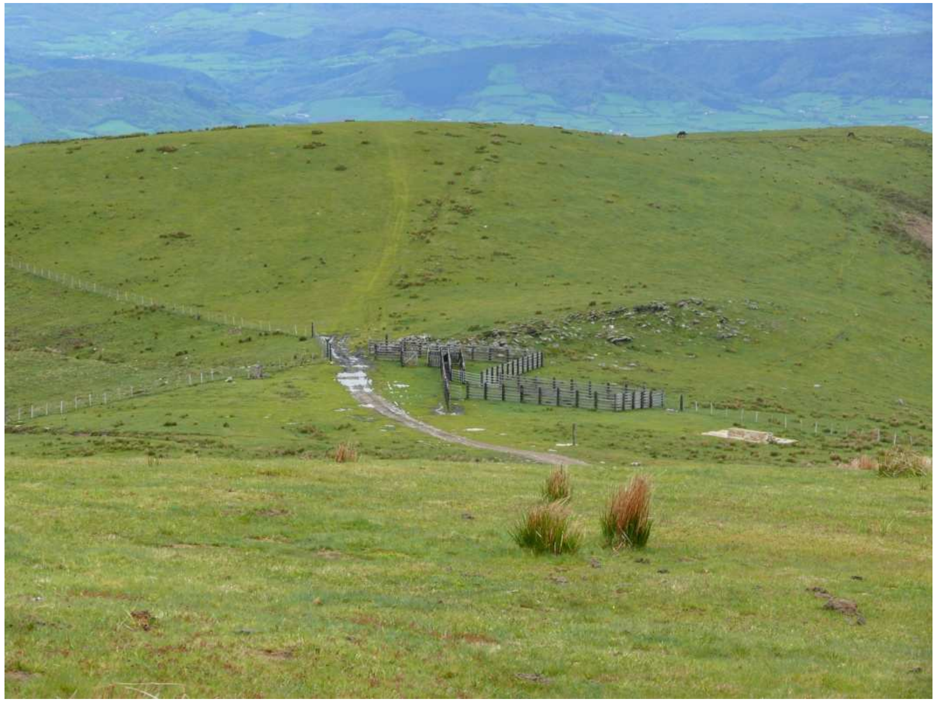

After measuring each indicator, we have to compare our results with the quantitative interpretation values provided in the AHC. In this way, we can find out to what qualitative category our results belong to: ***bad, medium*** **or** ***good*** health category. In addition, we must assign a score from 1 to 9 to our results **(indicator value)**.

Both basic and advanced indicators are grouped into a variety of ecosystem services delivered by healthy agroecosystems, in order to facilitate interpretation by decision-takers and, above all, provide stability to long-term monitoring programs against changes in methods, parameters, etc. The mean value of all indicators included in a particular ecosystem service indicates to what extent our agroecosystem can provide that specific ecosystem service **(ecosystem service value)**.

DETERMINATION OF AGROECOSYSTEM HEALTH

A value of **overall agroecosystem health** can be obtained by calculating the mean value of all measured ecosystem services. It must be taken into consideration that a healthy agroecosystem is expected to properly provide all the essential ecosystem services listed in the AHC. Therefore, if we obtain a low value for a specific ecosystem service, the agroecosystem cannot be claimed to have a good overall health, independently of the mean value obtained from all the ecosystem services included in the AHC.

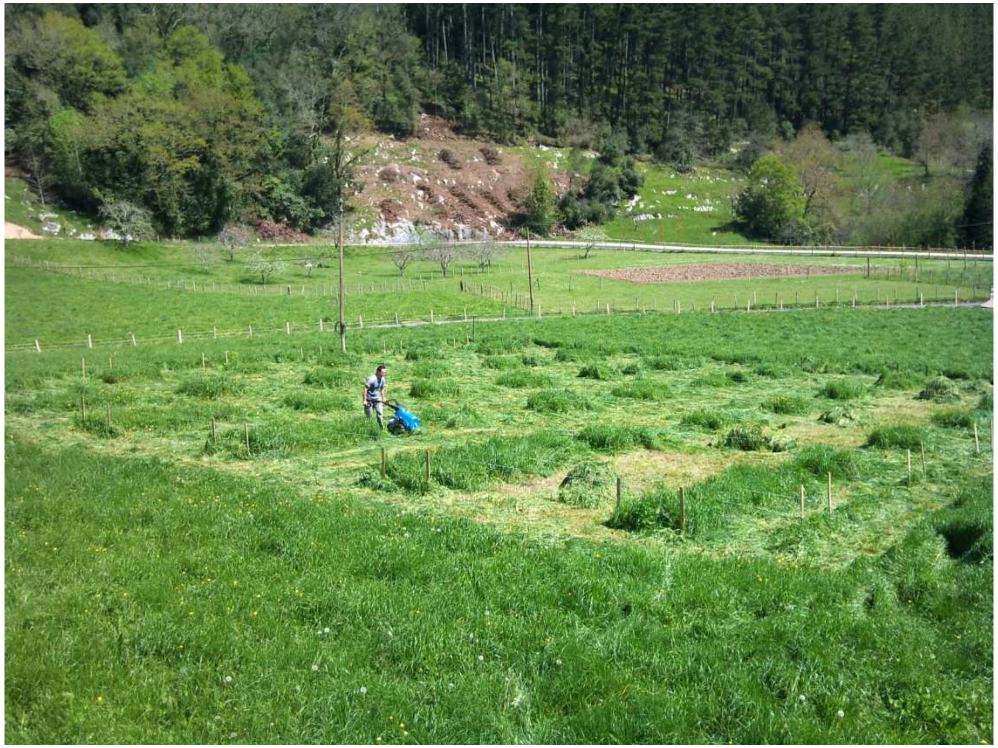

When all the indicators listed in the AHC cannot be measured, the same calculations can be performed. However, this might affect the reliability of the diagnosis.

MEASUREMENT OF INDICATORS

Year after year, indicators must be measured at the same time of the year (around the same month or season). When possible, measurements should be taken 2 to 5 days after a significant rainfall to avoid the soil being either too wet or too dry. It is important to avoid very hot or cold days, as this might affect the activity of soil organisms.

Make sure to always measure the indicators in the same way (same person, same technique, same hour of the day, etc.) to increase the reliability of the diagnosis. If different areas can be visibly identified in the study plot (in terms of vegetation, slope, humidity, etc.), they must be assessed separately.

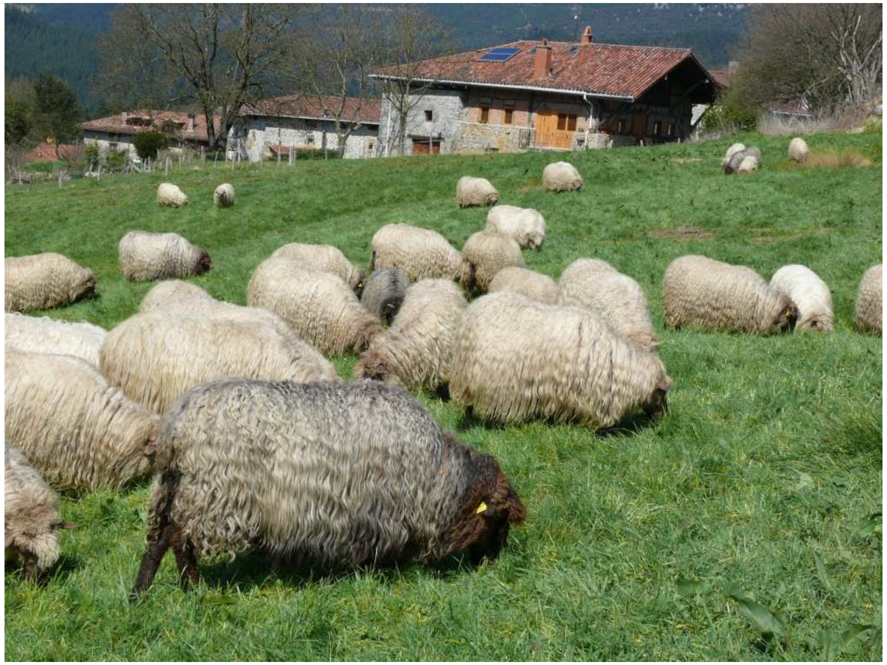

Don't be disheartened if, at the beginning, you get low scores in the AHC, as the values for the different indicators largely depend on the specific edaphoclimatic conditions present in your study plot. What is really important is to observe **temporal trends** (*i.e.*, how indicator values change from year to year). After a certain time, the application of good agricultural practices will lead to a better overall agroecosystem health, as reflected by higher scores in the AHC.

INDICATORS FOR A BASIC DIAGNOSIS

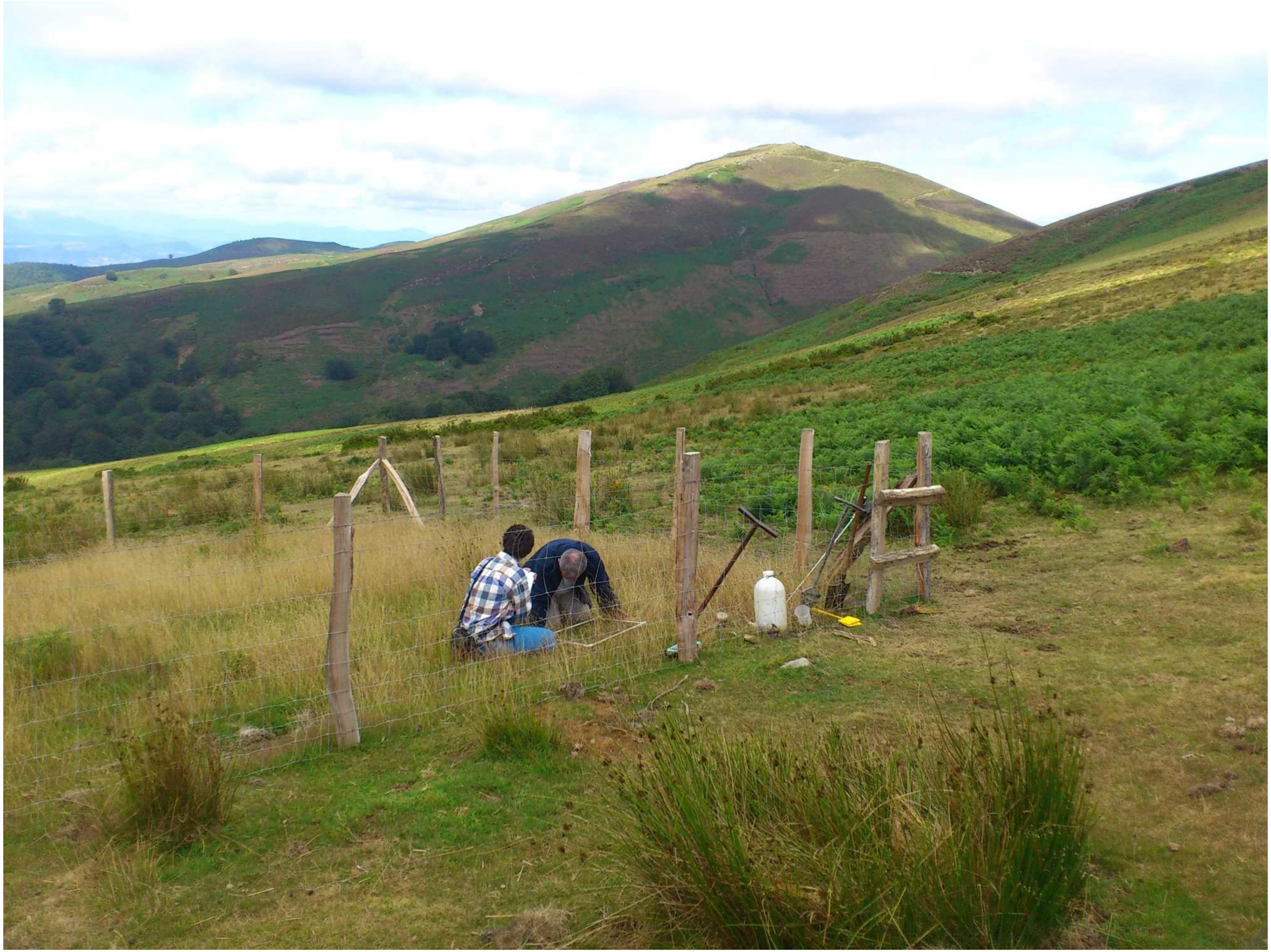

INDICATORS FOR A BASIC DIAGNOSIS

1. PASTURE PRODUCTION

1.1. Fresh weight (kg/m^2 per year)

Cut the vegetation in an area (0.5 m x 0.5 m = 0.25 m^2) not being used for grazing and then weigh it or, alternatively, keep it in a plastic bag (to avoid loss of humidity) until you can do so. Repeat the operation in four other areas within your study plot and then add up the weights (in kilograms).

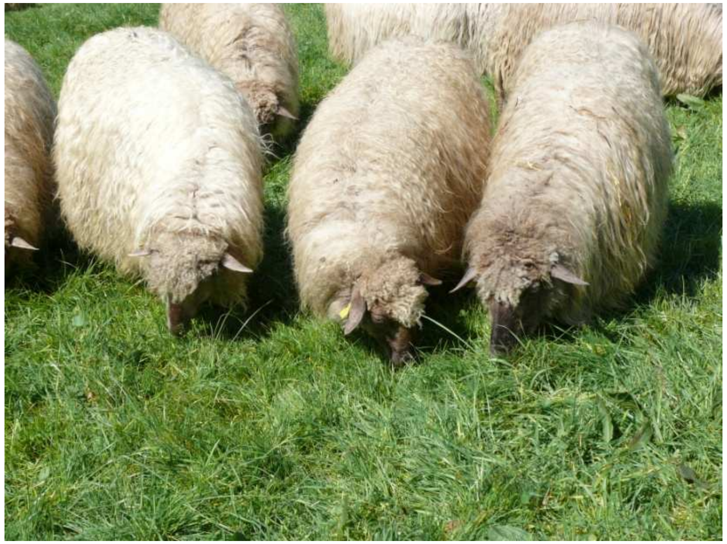

Carry out this procedure several times throughout the year (at least once every season; ideally, once a month to better simulate the effect of grazing).

Compare your results (kg/m^2 per year) with the quantitative reference values in the AHC and assign them a value from 1 to 9.

1.2. Animal rejection (%)

Visually estimate the percentage of grassland surface area (%) rejected by the grazing animals (*i.e.*, not being used as forage). Compare your results with the quantitative reference values in the AHC and assign them a value from 1 to 9.

2. CONSERVATION OF BIODIVERSITY

2.1. Number of plant species

Randomly* throw a 0.5 x 0.5 m square quadrat (a square wooden frame) within the study plot and then count the number of different plant species inside the quadrat (you don´t need to identify them, just count the number of different plant species). Throw the quadrat again and add up the number of new plant species you find. Repeat the operation until no new species appear (5-10 throws are normally needed). Finally, write down the total number of different plant species in your study plot.

Compare your results (number of plant species) with the quantitative reference values in the AHC and assign them a value from 1 to 9.

**Note: "throwing" a quadrat is not truly random, but a discussion on proper random sampling is outside the scope of this document.*

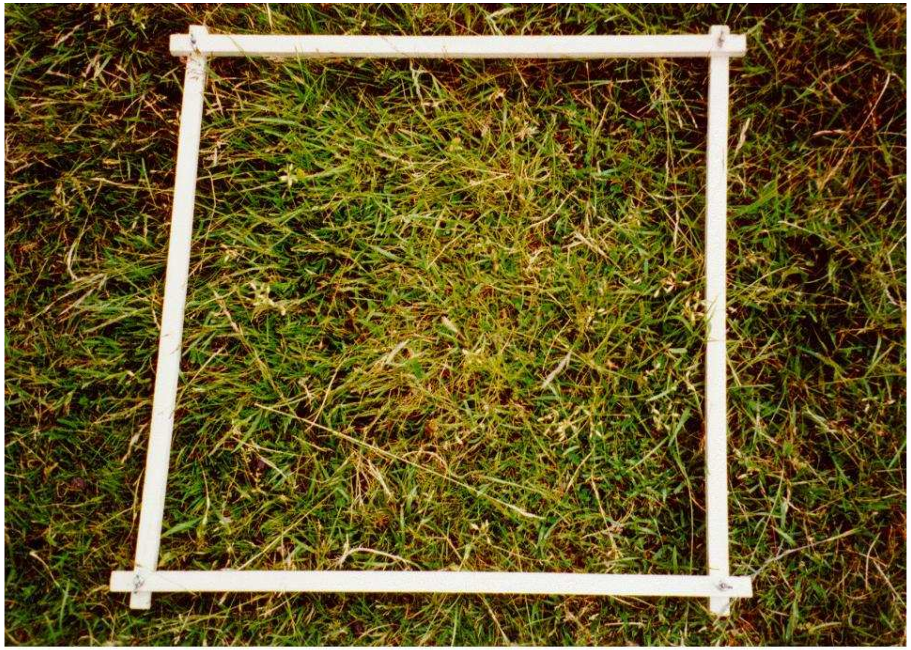

2.2. Number of plant strata

Visually identify whether 1, 2 or 3 plant strata (herbaceous, shrub and tree) are present in your study plot. Compare your results (number of plant strata) with the quantitative reference values in the AHC: 3 strata = 8 points, 2 strata = 5 points, 1 stratum = 2 points.

Shrubs and trees provide shelter for grazing animals, while creating new ecological niches and habitats above- and belowground.

2.3. Number of types of macrofauna

Use a flat shovel to extract a block of soil: 30 cm deep x 25 cm long x 25 cm wide. Try to do so in less than one minute to prevent the escape of macrofaunal animals from the block. Count the different types of macrofaunal animals (not the number of individuals) present in the block: to this aim, first examine the surface of the block and then crumble it by hand. Use the pictures provided to facilitate identification. Repeat the operation three more times. Finally, calculate the mean value (number of types of macrofaunal animals), compare it with the quantitative reference values in the AHC, and assign it a value from 1 to 9.

Soil macrofauna is involved in degrading organic matter and mineralizing nutrients, controlling pathogen populations, improving and maintaining soil structure, and mixing organic matter through the soil.

TYPES OF MACROFAUNA

1. Worms (Oligochaeta)
2. Cockroaches (Dictyoptera)
3. Woodlice (Isopoda)
4. Millipedes (Diplopoda)
5. Centipedes (Chilopoda)

6. Earwigs (Dermaptera)
7. Ants (Hymenoptera)
8. Termites (Isoptera)
9. Grasshoppers (Orthoptera)
10. Beetles (Coleoptera)

11. True bugs (Heteroptera)
12. Spiders (Arachnida)
13. Snails (Gasteropoda)
14. Cicadas (Homoptera)
15. Others (Larvae, etc.)

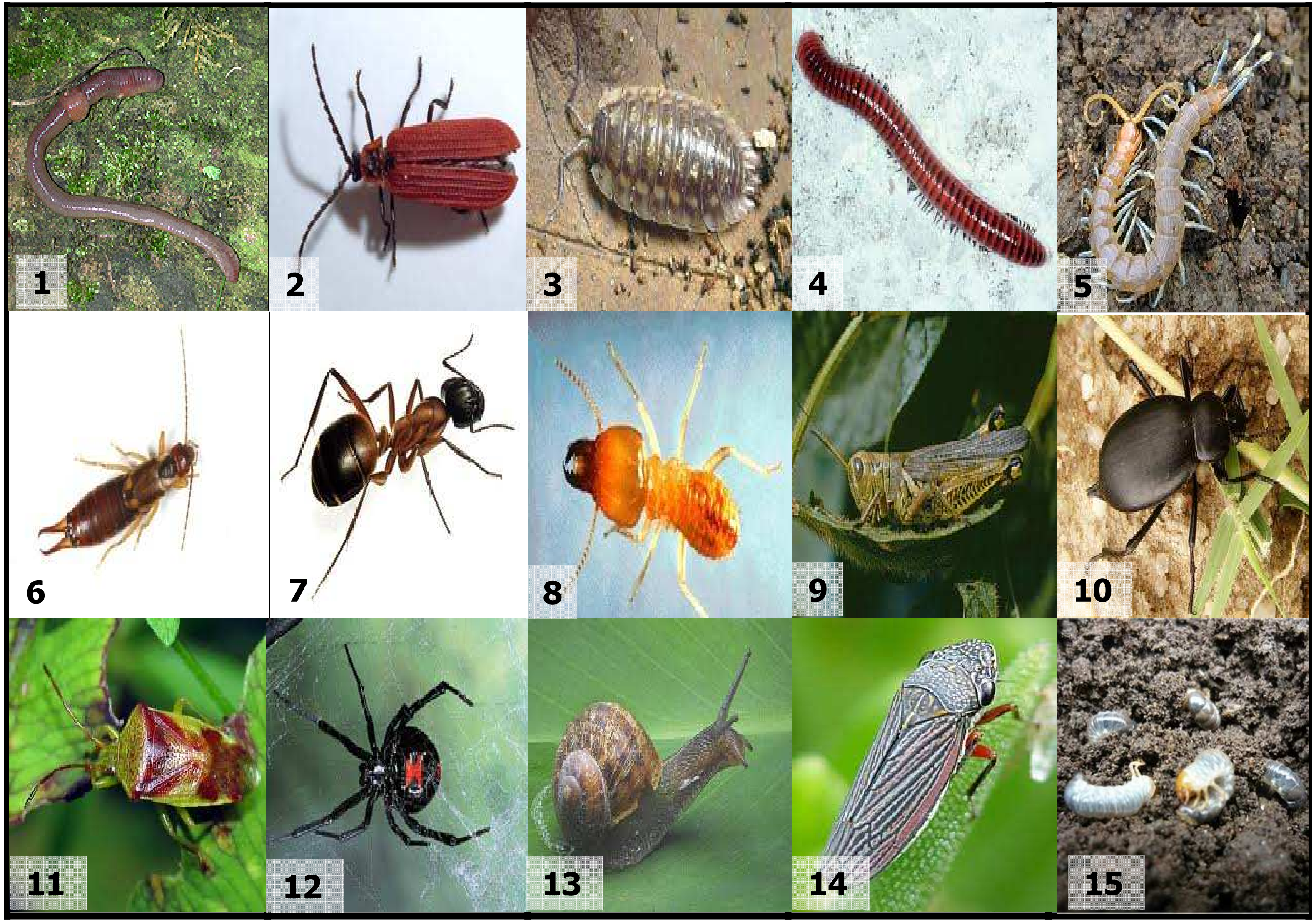

2.4. Number of invasive species

Look for plant and animal species considered invasive in your region. Compare your results (number of invasive species) with the quantitative reference values in the AHC and assign them a value from 1 to 9. Below we include some pictures of plant invasive species in our region.

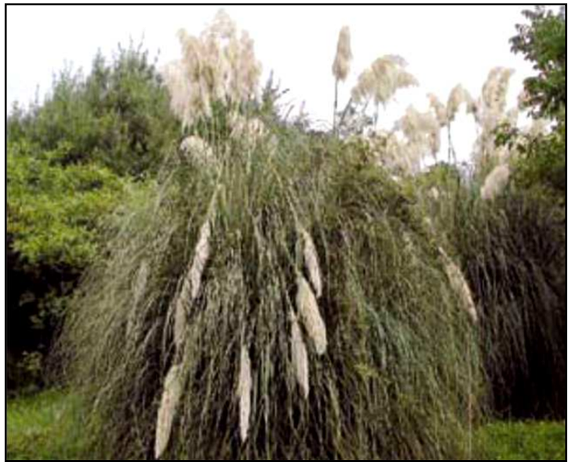

Cortaderia selloana

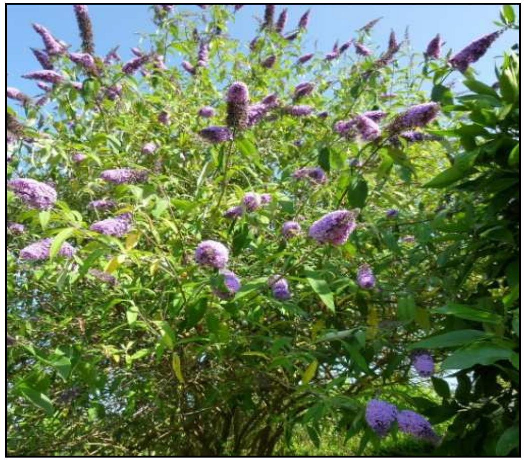

Buddleja davidii

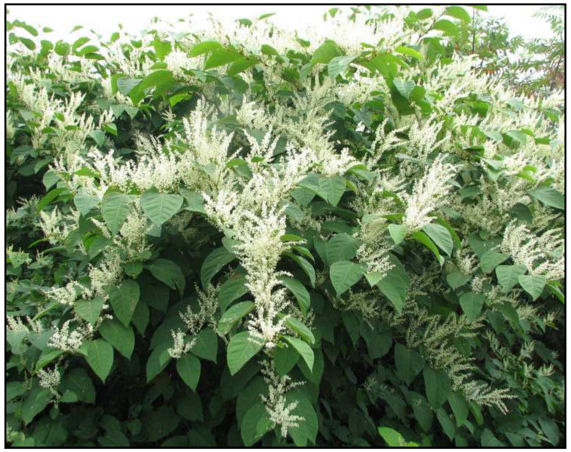

Fallopia japonica

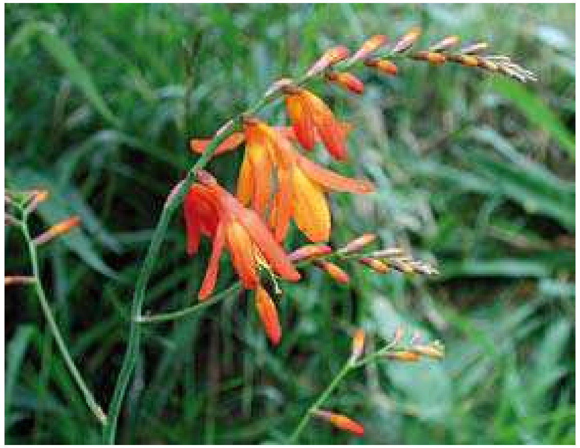

Crocosmia x crocosmiflora

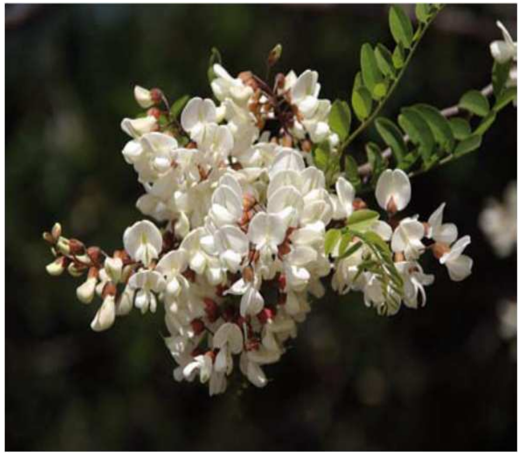

Robinia pseudoacacia

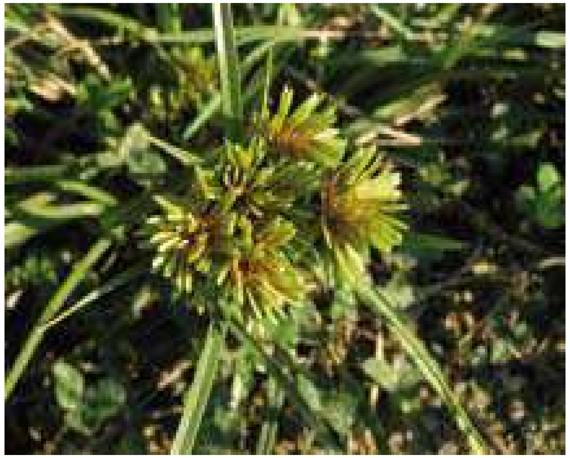

Cyperus eragrostis

3. SOIL CONSERVATION

3.1. Number of worms/m²

Use a flat shovel to extract a block of soil: 30 cm deep x 25 cm long x 25 cm wide. Try to do so in less than one minute to prevent the escape of macrofaunal animals from the block. Count the number of worms (individuals) present in the block. Repeat the operation 3 more times. After counting the number of worms in the four blocks, add the values up and, then, multiply that number by 4 (to obtain the number of worms per square metre). Compare your results (number of worms/m^2) with the quantitative reference values in the AHC and assign them a value from 1 to 9.

Worms prefer neutral soils rich in organic matter, where they favour the penetration of roots, water and air through their tunnel networks, thus reducing the adverse effects of soil compaction.

3.2. Compaction

Two measurements must be taken for this indicator:

- *Penetrability* (cm): take a corrugated rod (1 m long, 8 mm diameter) such as those used in building construction. Push it in the soil as much as you can with a modest effort and then calculate the soil depth (cm) reached. If you hit a stone, try again. Repeat the operation three more times. Finally, calculate the mean value, compare it with the quantitative reference values in the AHC, and assign it a value from 1 to 9.

- *Root depth* (cm): use a flat shovel to extract a block of soil: 30 cm deep x 25 cm long x 25 cm wide. Repeat the operation three more times. In the soil profile, measure the maximum depth (cm) reached by a significant number of roots. Finally, calculate the mean value, compare it with the quantitative reference values in the AHC, and assign it a value from 1 to 9.

3.3. Erosion risk (% bare soil)

Visually estimate the percentage of bare soil (no vegetation) in your study plot. Compare the result (%) with the quantitative reference values in the AHC, and assign it a value from 1 to 9.

Bare soil is exposed to wind and water erosion. There is a higher risk of erosion in steep slopes.

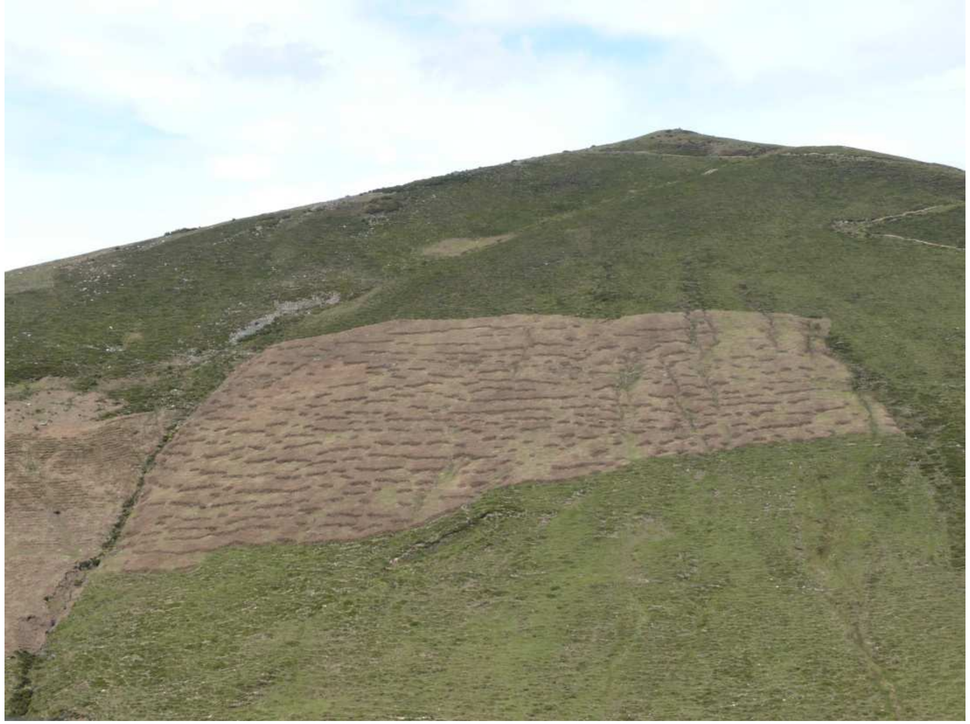

3.4. Infiltration capacity (minutes)

Take a galvanised steel tube (internal diameter: 15 cm), such as those used in building construction. First, cut a 10 cm long section off; then cut one of the ends slanting so that it will be easily inserted into the soil. Using a hammer and a block of wood, insert the tube in the soil to a depth of 2 cm, always avoiding stones, large roots, sticks and similar objects. Gently, pour 0.5 litres of water into the tube and wait until the water disappears (*i.e.*, until water percolates down the soil profile). Again, gently, pour another 0.5 litres of water and then write down the time it takes for the water to disappear. Repeat the operation at three more points within the study plot. Finally, calculate the mean value (minutes), compare it with the quantitative reference values in the AHC, and assign it a value from 1 to 9.

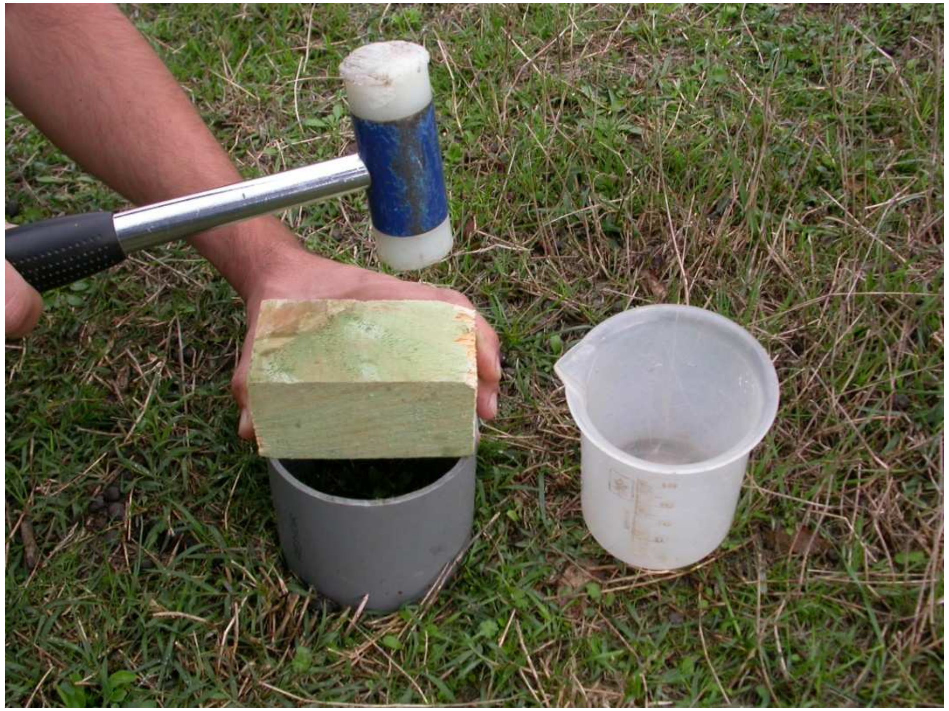

If it takes the water a long time to disappear, it means that the soil is prone to run-off and water erosion.

3.5. Plant colour

Visually check whether the vegetation in your study plot is, in general, pale green, dark green, or patchy in colour (a mosaic of green shades). Compare the result with the information provided in the AHC, and assign it a value from 1 to 9.

If not due to drought, a pale green-yellow colour may indicate a lack of certain soil nutrients (frequently, nitrogen).

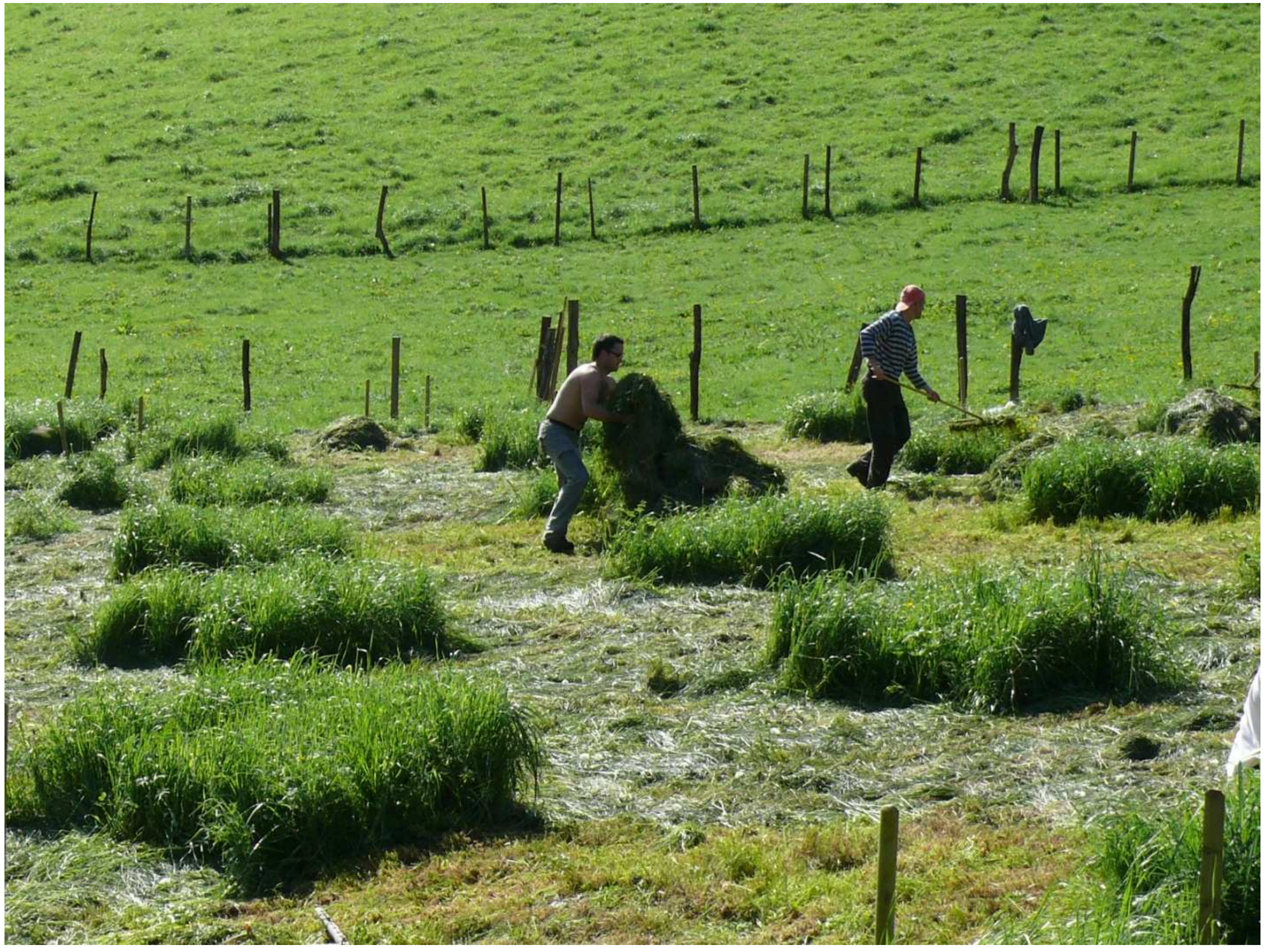

4. COMBATING CLIMATE CHANGE

4.1. Root abundance

Use a flat shovel to extract a block of soil: 30 cm deep x 25 cm long x 25 cm wide. Repeat the operation 3 more times. Visually estimate if root abundance in the four blocks can be regarded as low, medium or high. Compare the result with the information provided in the AHC, and assign it a value from 1 to 9.

In the rhizosphere (*i.e.*, the region of soil that surrounds, and is influenced by, plant roots), roots exude many substances into soil, including carbon-rich molecules. In addition, as roots age and die, they release different types and quantities of carbon substrates.

In the rhizosphere (*i.e.*, the region of soil that surrounds, and is influenced by, plant roots), roots exude many substances into soil, including carbon-rich molecules. In addition, as roots age and die, they release different types and quantities of carbon substrates.

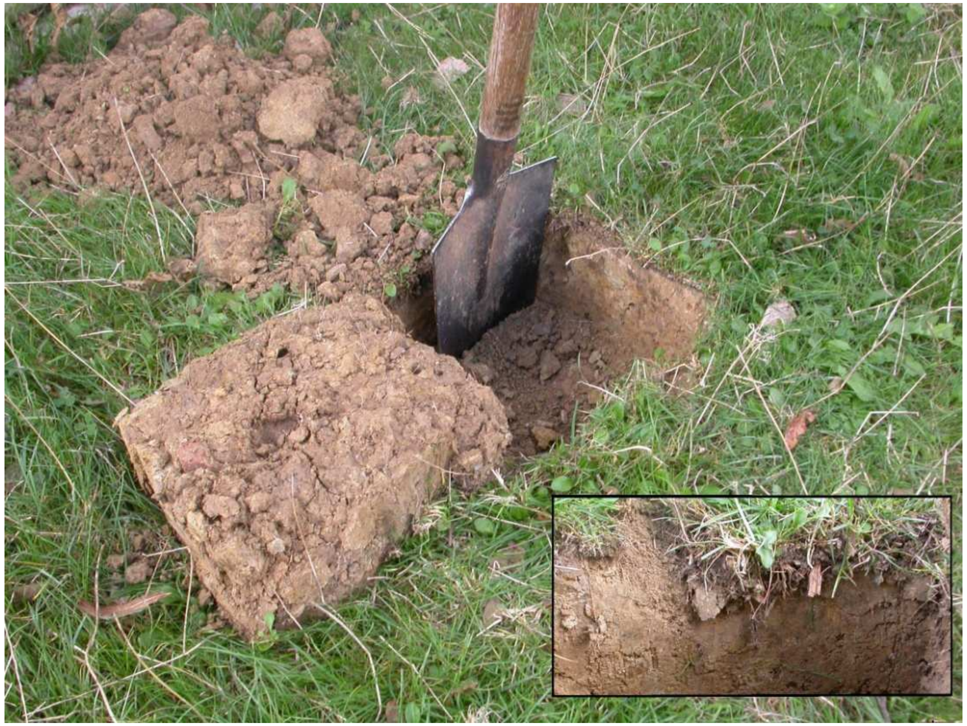

4.2. Soil colour

Use a flat shovel to extract a block of soil: 30 cm deep x 25 cm long x 25 cm wide. Repeat the operation 3 more times. Visually estimate if soil colour in the four blocks can be regarded as light, medium or dark. Compare the result with the information provided in the AHC, and assign it a value from 1 to 9.

Soils rich in organic material usually show a dark colour.

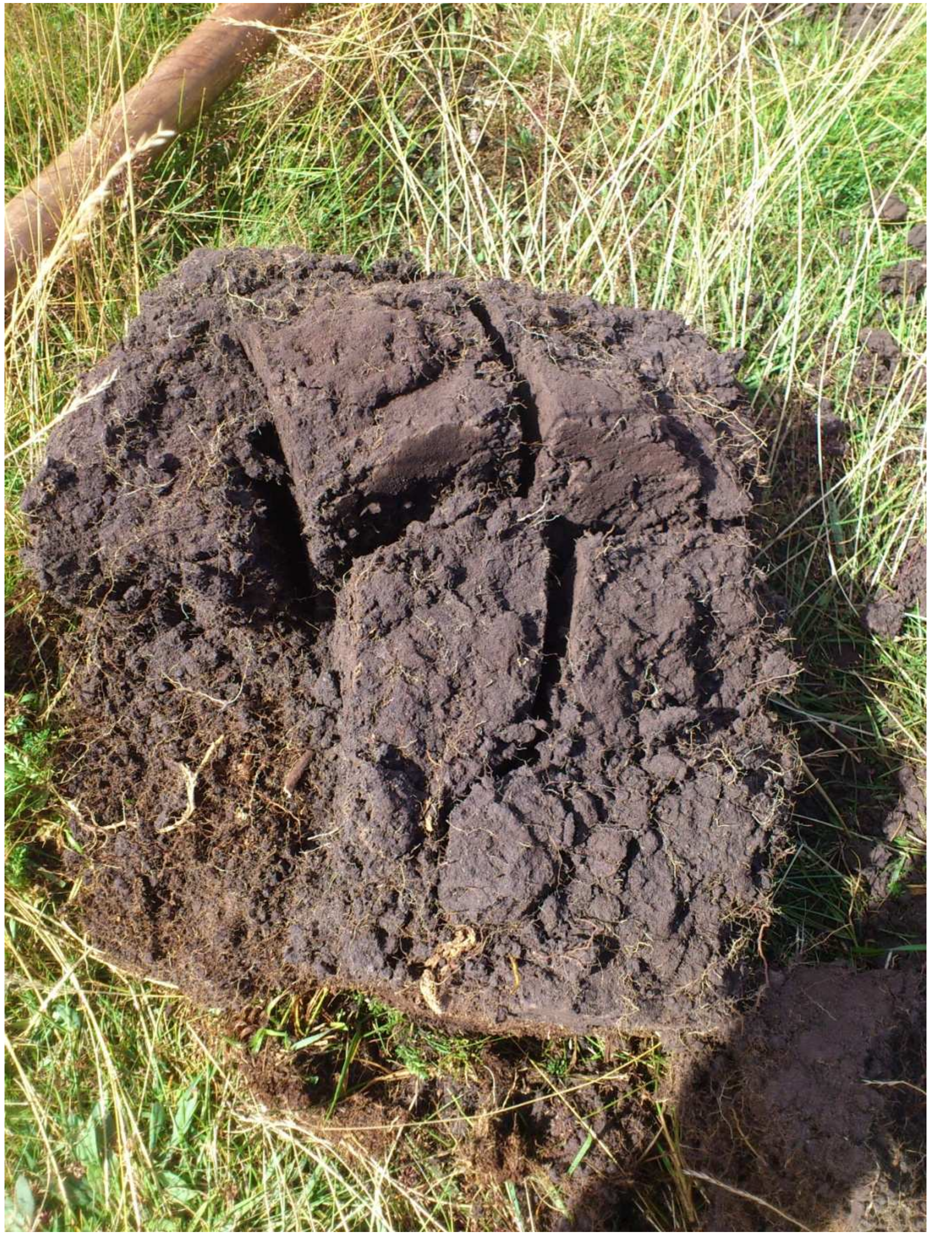

INDICATORS FOR AN ADVANCED DIAGNOSIS

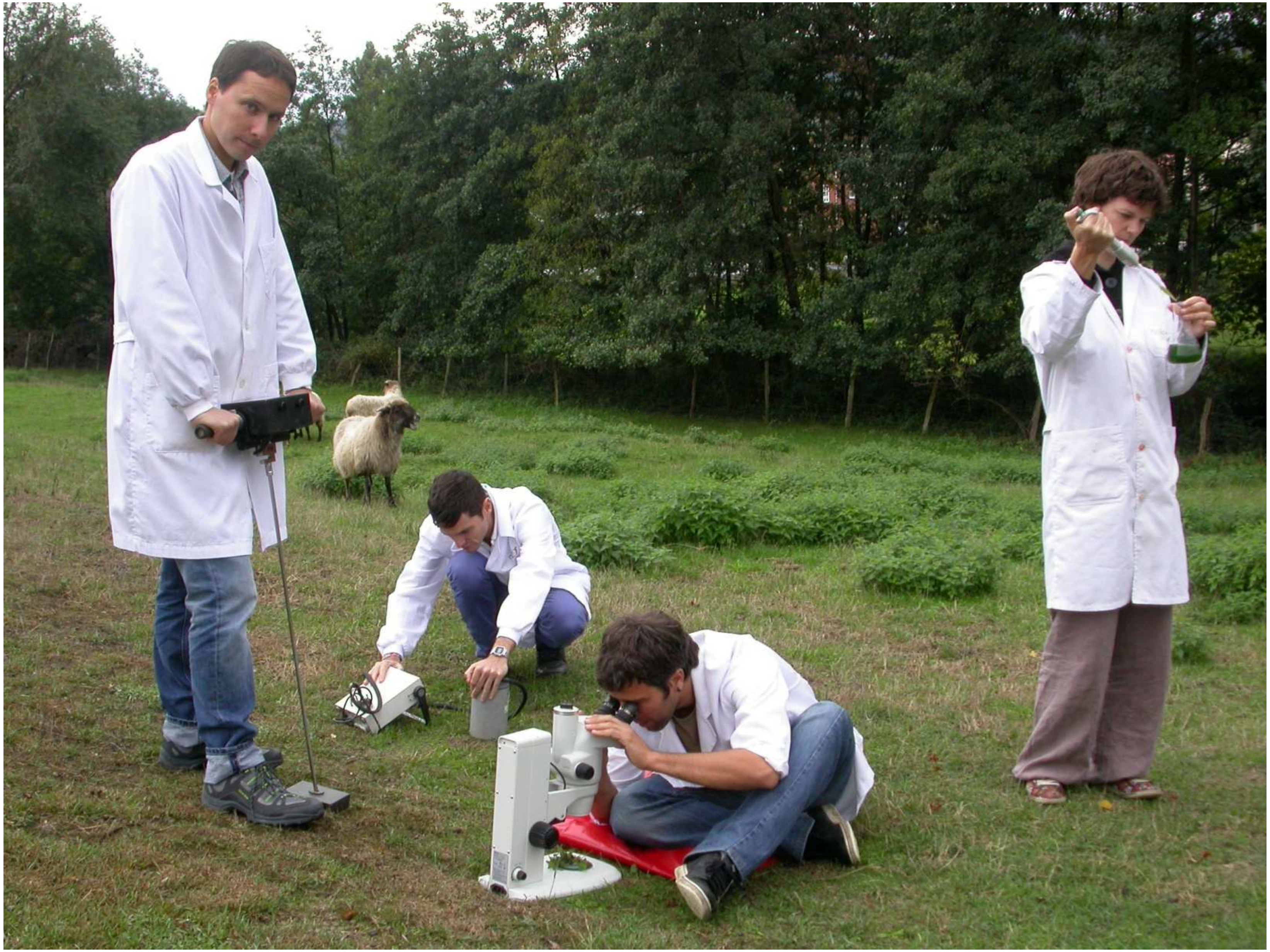

INDICATORS FOR AN ADVANCED DIAGNOSIS

1. PASTURE PRODUCTION

1.1. Dry weight (t/ha per year)

For the determination of dry weight, a representative sample of plant material is dried in an oven at 70 ^{o}C for 48 hours, and then weighed. The result, expressed in t/ha per year, is compared with the quantitative reference values in the AHC, and then a value from 1 to 9 is assigned.

2. CONSERVATION OF BIODIVERSITY

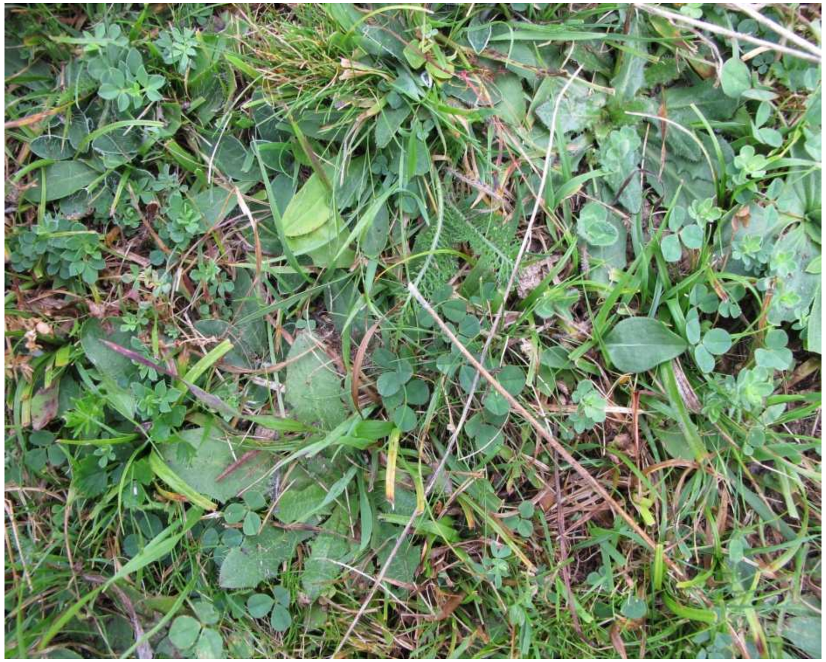

2.1. Plant diversity (Shannon´s index)

The number of plant species (richness) is calculated as previously described (see Basic Indicator 2.1). The percent cover (%) for each plant species present in the 0.5 x 0.5 square quadrats is also determined. These two values are used to calculate the Shannon´s diversity index (H') according to the formula $H' = -\Sigma\ p_i \times \log_b(p_i)$, p_i = proportion of species *i* relative to the total number of species. Compare your result with the quantitative reference values in the AHC and assign it a value from 1 to 9.

2.2. Mesofauna diversity (index)

A cylindrical soil sample (diameter = 10 cm, depth = 5 cm) is collected from the study plot. To extract the mesofauna from the soil sample, the Berlese-Tullgren extraction method is followed: put a 2 mm metal mesh (*e.g.*, a strainer) over a funnel, and place the soil sample on the metal mesh. Then, place the funnel beneath (20 cm away) a 50 watt bulb. Mesofauna organisms are collected in an alcohol-containing small jar located beneath the funnel.

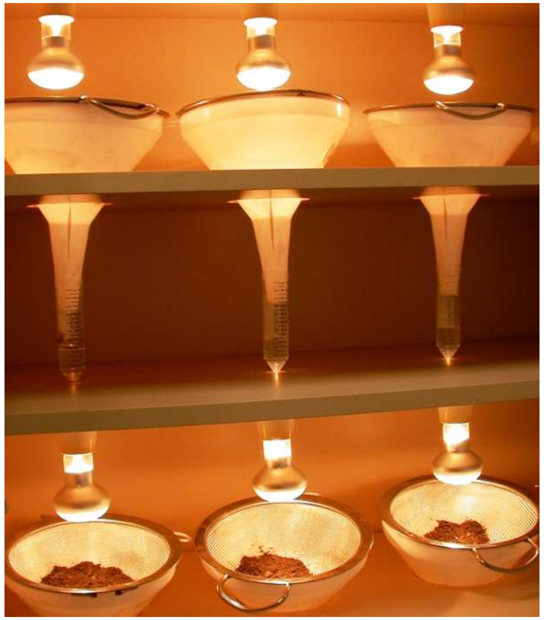

A magnifying glass and the pictures provided are used to identify the different types of mesofauna present in the soil sample (NOT to count the number of invividuals). As shown in the pictures, a numerical value (**see number in red colour**) is assigned to each type of mesofauna present in the sample (27 different types of mesofauna have been included). Add up all these values to obtain the mesofauna diversity index. Repeat the operation at three more points within the study plot. Calculate the mean value, compare it with the quantitative reference values in the AHC, and assign it a value from 1-9.

Mesofauna feed upon microorganisms, other soil animals, decaying plant or animal material, living plants, or fungi. Most of them feed on decaying plant material. They open drainage and aeration channels in the soil ecosystem by removing roots. These channels contain mesofaunal fecal material that can be broken down by smaller organisms.

TYPES OF MESOFAUNA

1.Protura 20

2.Diplura 20

3.Collembola 10

4. Microcoryphia 10

5.Zygentomata 10

6.Dermaptera 1

7.Orthoptera 10

8.Embioptera 10

9.Blattaria 5

10.Psocoptera 1

11.Hemiptera 5

12.Thysanoptera 1

13.Coleoptera 10

14.Hymenoptera 3

15. Diptera (L) 10

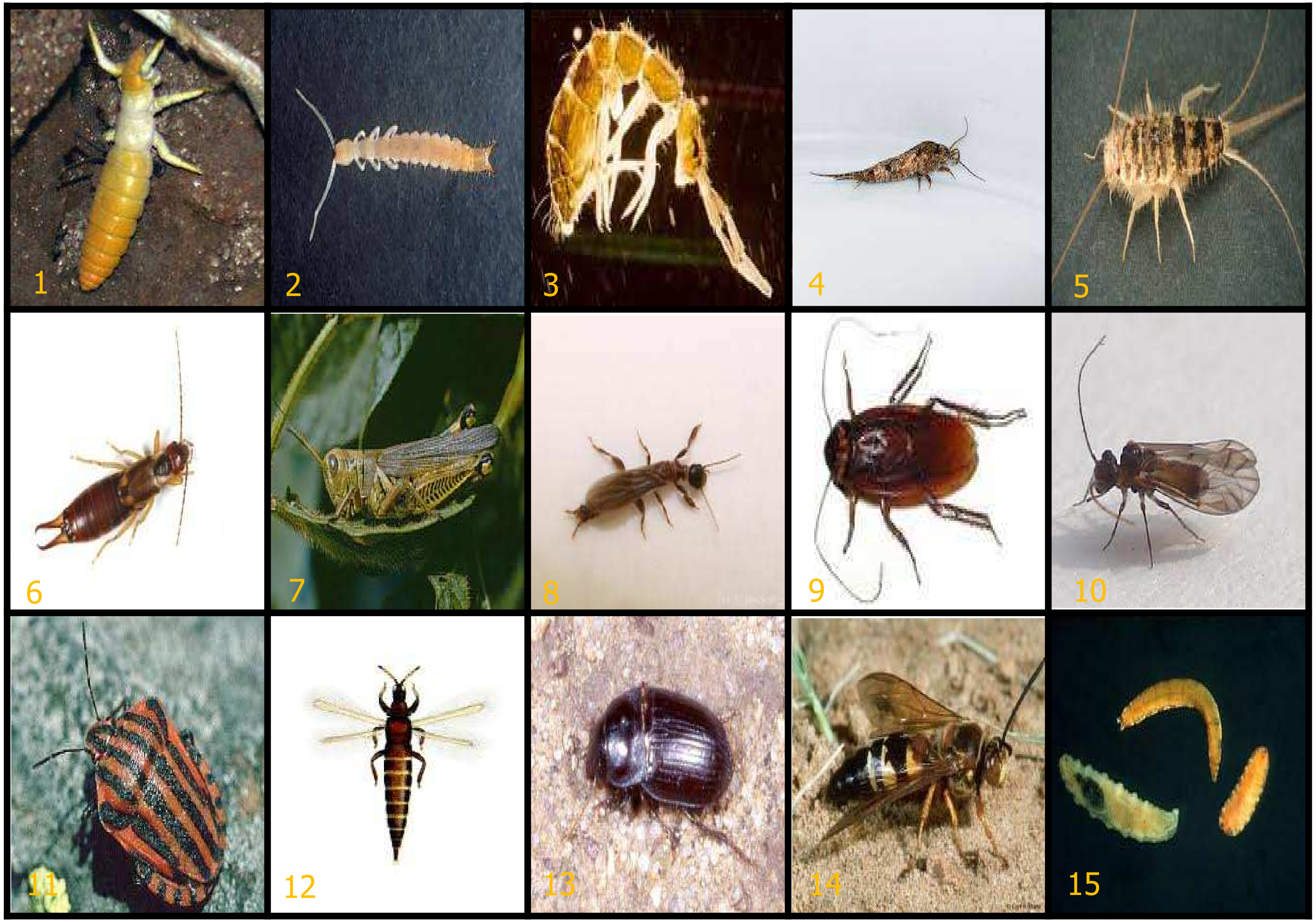

TYPES OF MESOFAUNA

16. Holometabolous(L)10

17. Holometabolous 1

18. Acari 20

19. Araneae 3

20. Opiliones 10

21. Palpigradi 20

22. Pseudoscorpion 20

23. Isopoda 10

24. Chiliopoda 15

25. Diplopoda 15

26. Pauropoda 20

27. Symphyla 20

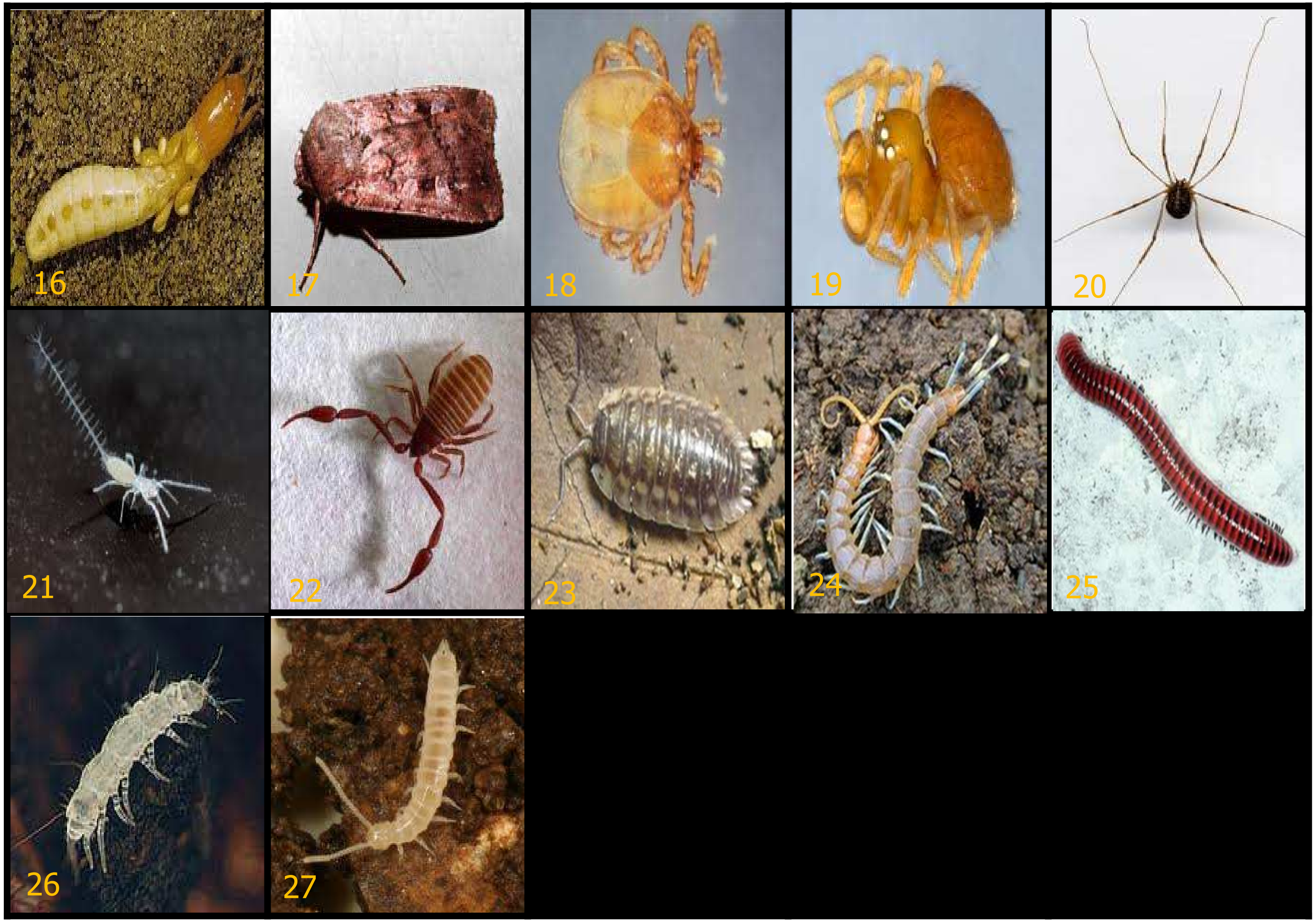

2.3. Fungal functional diversity (Shannon´s index)

Four soil samples are randomly collected within the study plot. Each sample consists of 10 subsamples of surface soil collected with a soil core sampler (diameter = 2.5 cm, depth = 10 cm). Samples are homogenised, sieved (2 mm mesh) and stored at 4 ºC until analysis. Samples must be analysed within one month of collection. For the calculation of fungal functional diversity, the procedure by Shugeng *et al*. (2009), using Biolog® FF MicroPlates, is followed, based on the capacity of the soil fungal community to use 95 different substrates as carbon source. Calculate the Shannon´s diversity index (H'), compare the result with the quantitative reference values in the AHC and assign it a value from 1 to 9.

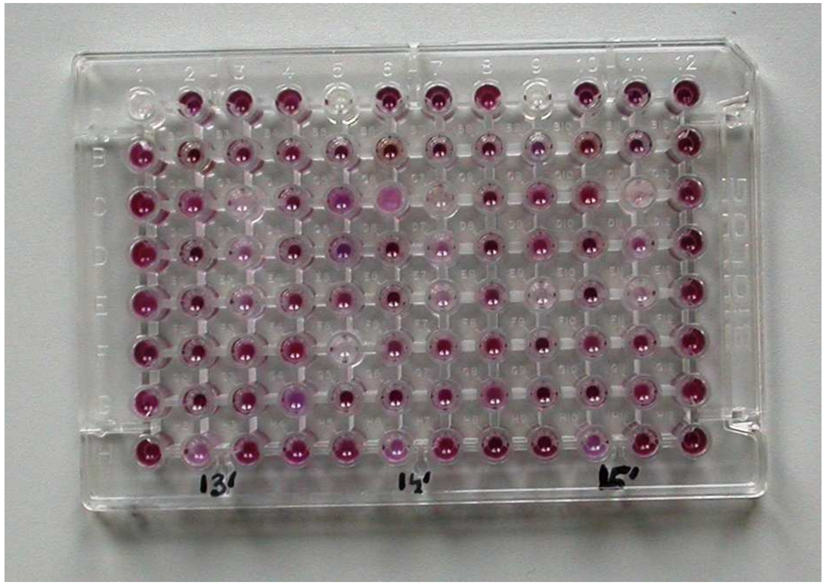

2.4. Bacterial functional diversity (Shannon´s index)

Sampling and processing is carried out as described in Advanced Indicator 2.3. For the calculation of bacterial functional diversity, the procedure by Mijangos *et al*. (2009), using Biolog® ECO Plates, is followed, based on the capacity of the soil bacterial community to use 31 different substrates as carbon source. Calculate the Shannon´s diversity index (H'), compare the result with the quantitative reference values in the AHC and assign it a value from 1 to 9.

2.5. Fungal genetic diversity (richness)

Sampling and processing is carried out as described in Advanced Indicator 2.3. For longer periods of storage time, samples can be stored frozen at -20 or -80 °C. For the calculation of fungal genetic diversity, the procedure by Epelde *et al.* (2012) is followed: DNA is extracted with the MoBio® Kit, amplified by PCR, and subjected to denatured gradient gel electrophoresis (DGGE). Calculate richness (number of bands), compare the result with the quantitative reference values in the AHC and assign it a value from 1 to 9.

2.6. Bacterial genetic diversity (richness)

Sampling and processing is carried out as described in Advanced Indicator 2.3. For longer periods of storage time, samples can be stored frozen at -20 or -80 °C. For the calculation of bacterial genetic diversity, the procedure by Mijangos *et al.* (2009) is followed: DNA is extracted with the MoBio® Kit, amplified by PCR, and subjected to denatured gradient gel electrophoresis (DGGE). Calculate richness (number of bands), compare the result with the quantitative reference values in the AHC and assign it a value from 1 to 9.

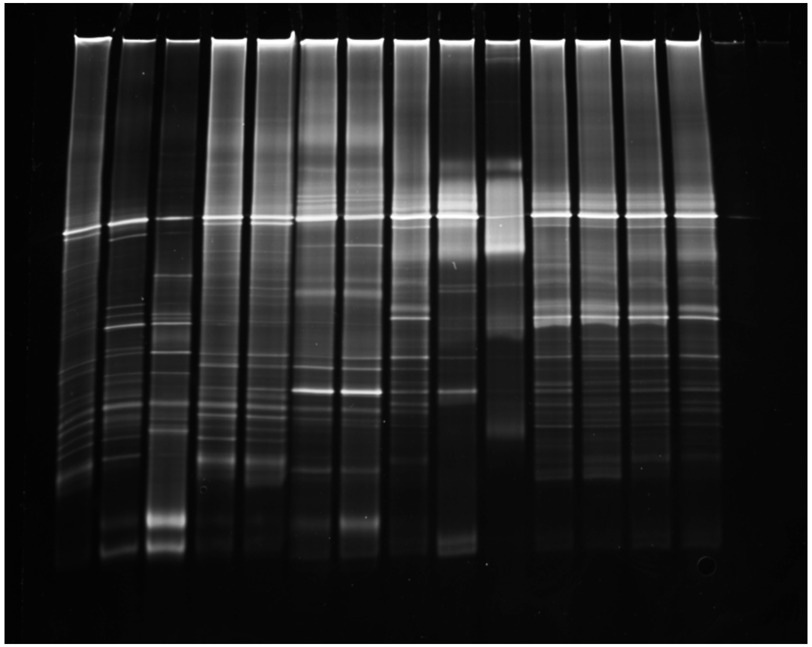

2.7. Total genetic diversity (Shannon´s index)

Sampling and processing is carried out as described in Advanced Indicator 2.3. For longer periods of storage time, samples can be stored frozen at -20 or -80 °C. For the determination of total genetic diversity, next generation sequencing techniques are used. Richness and abundance of operational taxonomic units is determined following Zinger *et al.* (2011). Calculate the Shannon´s diversity index (H'), compare the result with the quantitative reference values in the AHC and assign it a value from 1 to 9.

Soils are home to a prodigious diversity of life, which can often be several orders of magnitude greater than that present aboveground. Soil biodiversity is responsible for providing many of the ecosystem services on which human society relies.

3. SOIL CONSERVATION

3.1. Basal respiration: indicator of microbial activity

Sampling and processing is carried out as described in Advanced Indicator 2.3. For the determination of basal respiration, CO_2 emission rate from soil is measured in a hermetic jar incubated for 3 days at 30 ^{o}C and quantified by means of NaOH-titration (ISO 16072, 2002). The result, expressed in mg C-CO_2/kg per hour, is compared with the quantitative reference values in the AHC and then a value from 1 to 9 is assigned.

High soil respiration rates are indicative of high biological activity (basal respiration values reflect the level of microbial activity, soil organic matter content and its decomposition). On the other hand, it must be taken into consideration that CO_2 released by soil respiration is a greenhouse gas.

3.2. Substrate-induced respiration: indicator of microbial biomass

Sampling and processing is carried out as described in Advanced Indicator 2.3. For the determination of substrate-induced respiration, CO_2 emission rate from soil is measured, in a hermetic jar incubated for 6 hours at 30 ^oC, immediately after the addition of a nutrient solution consisting of a mixture of glucose, KH_2PO_4 and $(NH_4)_2SO_4$, as described in ISO 17155 (2002).

Soil microbial biomass is the main driving force in the decomposition of organic materials and is frequently used as an early indicator of changes in soil properties resulting from soil management and environmental stresses in agricultural ecosystems.

3.3. Metabolic quotient

The metabolic quotient (qCO_2) is calculated as the ratio of basal respiration to substrate-induced respiration. The result is compared with the quantitative reference values in the AHC and then a value from 1 to 9 is assigned.

This ratio shows how efficiently heterotrophic microorganisms can transform organic carbon into microbial biomass. The ratio can be used as a stress indicator because different stress factors, such as heavy metal contamination and nutrient deficiency, increase qCO_2 values due to a decrease in microbial biomass and an increase in respiration rate.

3.4. Compaction (MPa)

For the determination of soil compaction, a digital penetrometer (Rimik CP40II) is used to quantify the pressure (MPa) needed to penetrate the soil. Four points are randomly selected within the study plot for this measurement. Calculate the mean value (MPa) of resistance to penetration in the upper 0-30 cm of the soil, compare it with the quantitative references values in the AHC, and then assign it a value from 1 to 9.

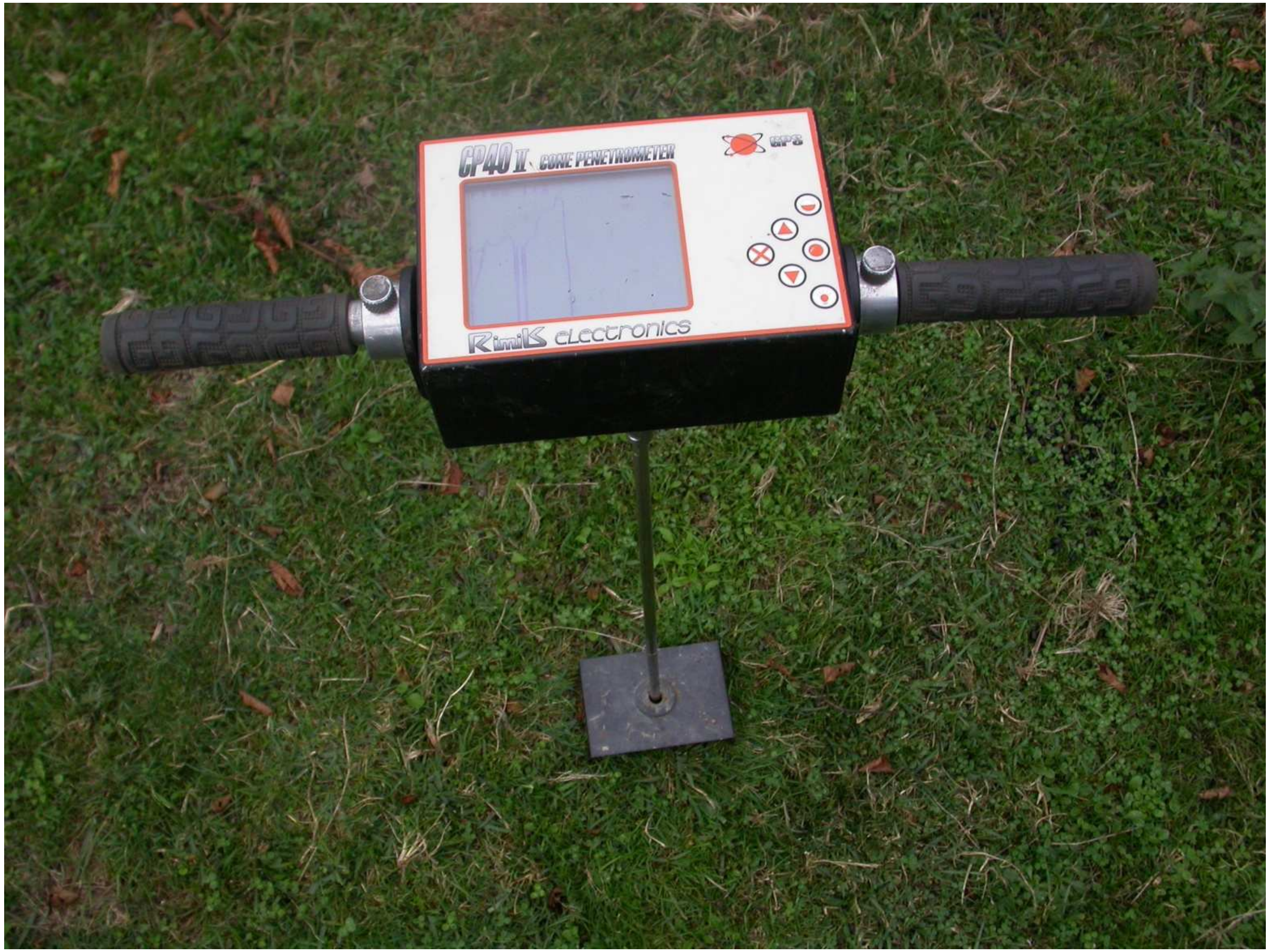

3.5. Soil-Acidity

Two measurements must be taken for this indicator:

- *Aluminum saturation (%):* sampling and processing is carried out as described in Advanced Indicator 2.3; however, in this case, soil samples can be stored at ambient temperature. Percent aluminum saturation can be determined following Standard Methods (MAPA, 1994). The result is compared with the quantitative reference values in the AHC and then a value from 1 to 9 is assigned.

"Percent aluminum saturation" is an expression that describes the relative abundance of aluminum in the soil: in particular, it describes the percent of the soil cation exchange capacity (CEC) occupied by exchangeable Al^{3+}.

Percent aluminum saturation of the soil ion exchange complex is a useful index for predicting the effects of aluminum toxicity on crop yield and root growth.

- *Soil pH:* sampling and processing is carried out as described in Advanced Indicator 2.3; however, in this case, soil samples can be stored at ambient temperature. The soil pH can be measured in water (1:2.5), as described in MAPA (1994). The result is compared with the quantitative reference values in the AHC and then a value from 1 to 9 is assigned.

In rainy areas, mountain pasture soils are typically acidic, due to intense leaching of bases. This acidification can limit plant growth and soil microbial activity.

3.6. Soil nitrogen

Sampling and processing is carried out as described in Advanced Indicator 2.3; however, in this case, soil samples can be stored at ambient temperature. Total nitrogen content (Kjeldahl method) can be measured following Standard Methods (MAPA, 1994). The result (%) is compared with the quantitative reference values in the AHC and then a value from 1 to 9 is assigned.

Nitrogen is a well-known essential macronutrient for plants and soil microorganisms. The quantity of nitrogen in soil is intimately associated with the level of soil organic matter. Legumes convert atmospheric N_2 to plant available forms via a symbiotic biological process involving *Rhizobium* bacteria and plant roots.

Nitrogen is frequently a growth-limiting plant nutrient; in consequence, there is often a very large crop-yield response to additional nitrogen.

On the other hand, nitrogen in the nitrate form is very soluble and one of the most mobile plant nutrients in soil; therefore, nitrate can easily be lost from soil and become a contaminant in surface or groundwater.

3.7. Soil phosphorus

Sampling and processing is carried out as described in Advanced Indicator 2.3; however, in this case, soil samples can be stored at ambient temperature. Olsen phosphorus content can be measured following Standard Methods (MAPA, 1994). The result (ppm) is compared with the quantitative reference values in the AHC and then a value from 1 to 9 is assigned.

Phosphorus is an essential macronutrient (plants require relatively large amounts of phosphorus) and one of the three nutrients generally added to soils in fertilizers.

Mismanagement of soil phosphorus can pose a threat to water quality: phosphorus lost from agricultural soil can contaminate lakes and rivers, leading to eutrophication.

3.8. Soil potassium

Sampling and processing is carried out as described in Advanced Indicator 2.3; however, in this case, soil samples can be stored at ambient temperature. Extractable potassium content can be measured following Standard Methods (MAPA, 1994). The result (ppm) is compared with the quantitative reference values in the AHC and then a value from 1 to 9 is assigned.

Potassium is an essential macronutrient for plant growth and soil microorganisms. For each soil and farming system there is an optimum level for soil potassium reserves. The application of potassium in manures and fertilizers should aim to maintain this optimum level.

4. COMBATING CLIMATE CHANGE

4.1. CO_2 emission from soil

This indicator can be measured *in situ* using a portable equipment (IRGA EGM-4, PP Systems^R). Four points are randomly selected within the study plot; at each point, a mean value of CO_2 emission (g CO_2/m^2 per hour) is obtained from five individual measurements. Calculate the mean value for the four abovementioned points, compare it with the quantitative references values in the AHC, and then assign it a value from 1 to 9.

CO_2 emissions from soil into the atmosphere contribute to climate change.

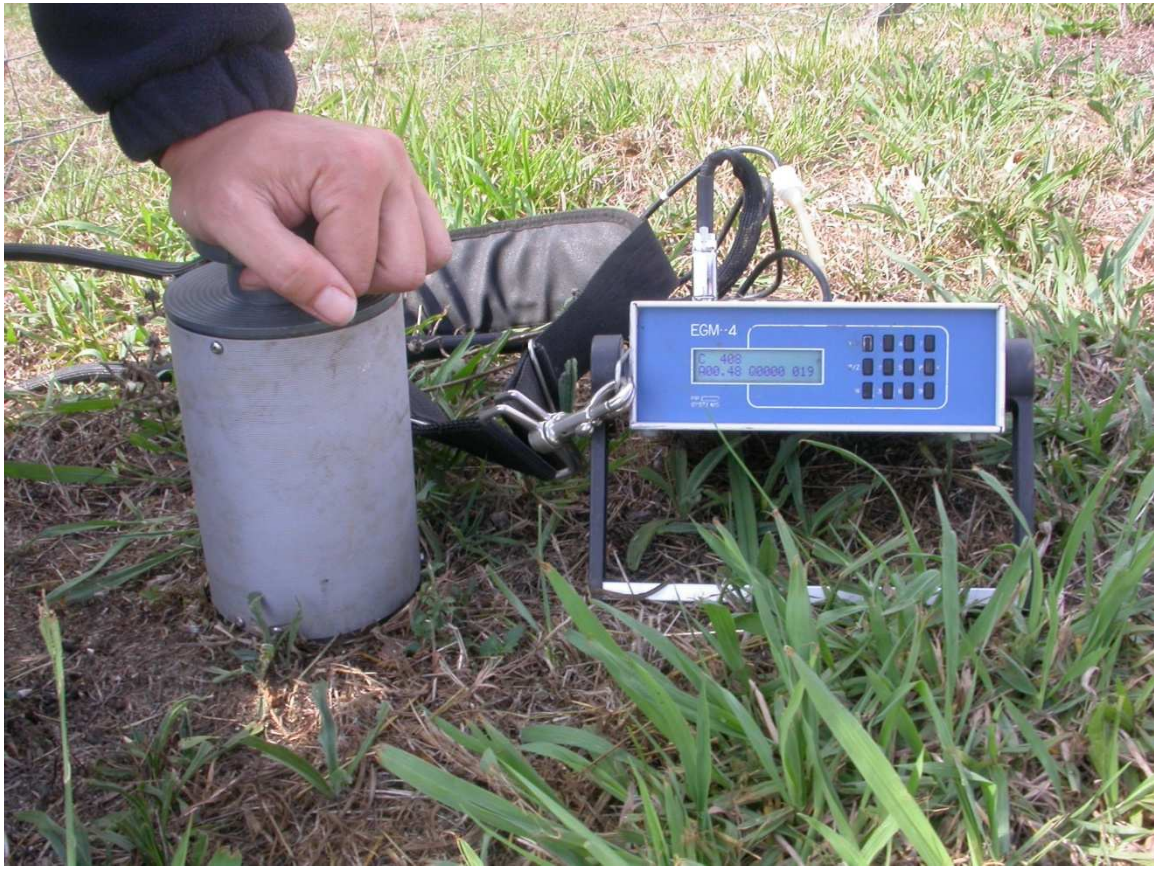

4.2. Soil organic matter

Sampling and processing is carried out as described in Advanced Indicator 2.3; however, in this case, soil samples can be stored at ambient temperature. The amount of oxidizable organic matter in soil can be measured following Standard Methods (MAPA, 1994). The result (%) is compared with the quantitative reference values in the AHC and then a value from 1 to 9 is assigned.

Managing soils to enhance carbon absorption from the atmosphere and its storage as soil organic matter is a promising option for attenuating global warming.

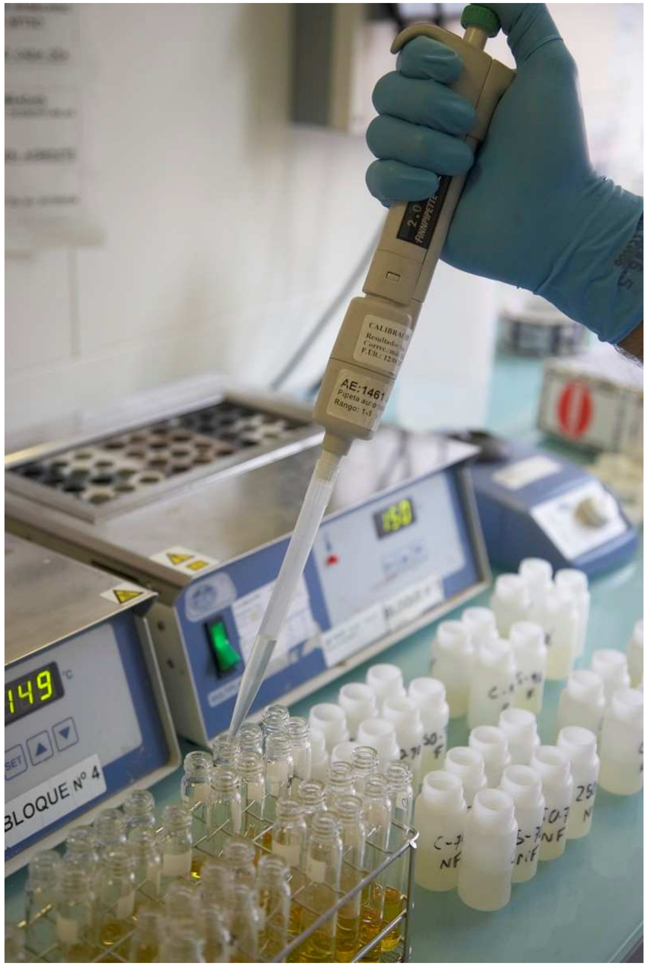

BAD RESULTS: WHAT DO THEY MEAN? HOW CAN I IMPROVE THEM?

ECOSYSTEM SERVICES	BAD RESULTS	REASONS/CONSEQUENCES	RECOMMENDATIONS
Pasture production	Low pasture production	Infertile soil<br>Inappropriate pasture management	Apply fertilizer and/or lime<br>Decrease livestock load
	High % animal rejection	Abundance of unpalatable and/or toxic plant species	Eliminate rejected species
Biodiversity conservation	Low number of plant species	Loss of plant richness	Reseed with valuable plant species
	Low number of plant strata	Loss of plant diversity, poor vegetation structure	Plant and protect trees and shrubs
	Low number of macrofauna types	Loss of animal diversity<br>Trophic chain negatively affected	Organic amendments<br>Increase plant cover
	Low number of mesofauna types	Loss of animal diversity<br>Trophic chain negatively affected	Organic amendments<br>Increase plant cover
	Presence of invasive species	Threat to autochthonous species	Erradicate invasive species
	Low microbial functional diversity	Loss of microbial diversity<br>Poor soil functioning	Organic amendments<br>Increase plant diversity and cover
	Low microbial genetic diversity	Loss of microbial diversity<br>Low soil resilience	Organic amendments<br>Increase plant diversity and cover
Soil conservation	Low number of worms	Soil acidification, soil compaction	Apply lime, organic amendments
	High soil compaction	Impaired root development	Decrease livestock load, organic amendments
	High percentage of bare soil	Overgrazing, increased erosion	Decrease livestock load, organic amendments
	Low infiltration capacity	Run-off, increased erosion, waste of water resource	Decrease livestock load, organic amendments
	Vegetation: pale green, patchy in colour	Lack of water, nitrogen or other nutrients	Apply fertilizer, organic amendments
	Low microbial activity and/or abundance	Soil acidification, contaminants, lack of water or nutrients	Apply lime, remediation, organic amendments
	High value of the metabolic quotient	Immature soil, contaminants, stressed communities	Organic amendments, remediation
	Low or high soil pH	Plant growth limited, microbial growth limited	Apply lime or sulphur, respectively
	Low N, P and/or K content	Plant growth limited, microbial growth limited	Apply fertilizer, organic amendments
	High N, P and/or K content	Eutrophication	Do not fertilize, decrease livestock load
Combating climate change	Low value of root abundance	Lower carbon sequestration	No tillage, apply fertilizer and/or lime
	Light coloured soil	Low carbon content in soil	No tillage, organic amendments
	High CO_2 emissions	CO_2 released to atmosphere	No tillage
	Low organic matter content	Low carbon content in soil	No tillage, organic amendments

Basic Health Diagnosis

Plot name:____ Date:__________________________

ECOSYSTEM SERVICES	BASIC INDICATORS	Bad 1..2..3	Medium 4..5..6	Good 7..8..9	Indicator value (1-9)	Service value (1-9)
PASTURE PRODUCTION	1.1. Fresh weight (kg/m^2 per year):<br>- mountain<br>- valley	<0.8<br><2	0.8-1,1<br>2-2.8	>1.1<br>>2.8		
	1.2. Animal rejection (%)	>25	5-25	<5		
BIODIVERSITY CONSERVATION	2.1. Plant species (number)<br>- mountain<br>- valley	<15<br><15	16-30<br>16-25	>30<br>>25		
	2.2. Plant strata (number)	1	2	3		
	2.3 Types of macrofauna (number)	<3	3-6	>6		
	2.4. Invasive species (number)	>1	1	0		
SOIL CONSERVATION	3.1. Worms (number/m^2)	<16	17-64	>65		
	3.2. Compaction - penetrability (cm)<br>3.2. Compaction - root depth (cm)	<3<br><15	3-15<br>15-30	>15<br>>30		
	3.3. Erosion risk (% bare soil)	>15	5-15	<5		
	3.4. Infiltration capacity (min)	> 30	10-30	< 10		
	3.5. Plant colour	pale	patchy	dark		
COMBATING CLIMATE CHANGE	4.1. Root abundance	low	medium	high		
	4.2. Soil colour	light	medium	dark		
**BASIC DIAGNOSIS**						Final Mark

Advanced Health Diagnosis

Plot name:____ Date:___________________________________

ECOSYSTEM SERVICES	ADVANCED INDICATORS	Bad 1..2..3	Medium 4..5..6	Good 7..8..9	Indicator value (1-9)	Service value (1-9)
**PASTURE PRODUCTION**	1.1. Dry weight (t/ha per year):<br>- mountain<br>- valley	<3<br><5.4	3-4,2<br>5.4-7.6	>4.2<br>>7.6		
**BIODIVERSITY CONSERVATION**	2.1. Plant diversity (H’ index)<br>- mountain<br>- valley	<1.5<br><1.3	1.5-2.5<br>1.3-2.3	>2.5<br>>2.3		
	2.2. Mesofauna types (índex)	<40	40-70	>70		
	2.3. Fungal functional diversity (H’ index)	<3	3-4	>4		
	2.4. Bacterial functional diversity (H’ index)	<3	3-4	>4		
	2.5. Fungal genetic diversity (richness)	<5	5-11	>11		
	2.6. Bacterial genetic diversity (richness)	<10	10-18	>18		
	2.7. Total genetic diversity (H’ index)	<2	2-3	>3		
**SOIL CONSERVATION**	3.1. Microbial activity (mg C-CO_2/kg.h)	<0.6	0.6-1	>1		
	3.2. Microbial abundance (mg C-CO_2/kg.h)	<10	10-18	>18		
	3.3. Metabolic quotient- qCO_2	>0.1	0.1-0.06	<0.06		
	3.4. Compaction-penetrability (MPa)	> 3	2-3	<2		
	3.5. Acidity- aluminium saturation (%)<br>Acidity- pH	>20<br><5 or >7.5	10-20<br>5-5.9	<10<br>6-7.5		
	3.6. N total (%)	<0.10 or >3	0.11-0.29	0.3-3		
	3.7. Olsen P (ppm)	<8 or >45	8-15	15.1-45		
	3.8. Extractable K (ppm)	<80 or >300	80-120	121-350		
**COMBATING CLIMATE CHANGE**	4.1. CO_2 soil emissions (g CO_2/m^2. h)	>3	1.5-3	<1.5		
	4.2. Organic matter (%):<br>- mountain<br>- valley	<5<br><2	5-10<br>2-4	>10<br>>4		
**ADVANCED DIAGNOSIS**						Final Mark
